# The noncanonical function of liver-type phosphofructokinase potentiates the efficacy of HDAC inhibitors in cancer

**DOI:** 10.1038/s41392-025-02443-0

**Published:** 2025-10-14

**Authors:** Taiyu Shang, Tianyi Jiang, Jiangqi Tan, Haolin Jiang, Mengyou Xu, Yufei Pan, Yunkai Lin, Xiaowen Cui, Chenxi Tian, Huibo Feng, Yibin Chen, Mengmiao Pei, Xin Geng, Shuqun Cheng, Yexiong Tan, Hongyang Wang, Liwei Dong

**Affiliations:** 1 Fudan University Shanghai Cancer Center, Department of Oncology, Shanghai Medical College, Fudan University, Shanghai, China; 2 National Center for Liver Cancer, Naval Medical University, Shanghai, China; 3 International Cooperation Laboratory on Signal Transduction, Eastern Hepatobiliary Surgery Hospital, Shanghai, China; 4 Department of Oncology, Eastern Hepatobiliary Surgery Hospital, Shanghai, China; 5 Department of Surgery, Eastern Hepatobiliary Surgery Hospital, Shanghai, China; 6 State Key Laboratory of Oncogenes and Related Genes, Shanghai Cancer Institute, Renji Hospital, Shanghai Jiaotong University School of Medicine, Shanghai, China; 7 Shanghai Key Laboratory of Hepato-biliary Tumor Biology, Shanghai, China

**Keywords:** Predictive markers, Cancer therapy

## Abstract

Zinc-dependent histone deacetylases (HDACs) are pivotal enzymes governing the epigenetic modulation of gene expression through chromatin remodeling. The dysregulated expression of HDACs is intricately linked to various pathological conditions, including cancer and inflammation. Histone deacetylase inhibitors (HDACi) have shown therapeutic potential in certain hematologic malignancies. However, the clinical performance of HDACi in solid tumors remains unsatisfactory, and the precise mechanisms of its therapeutic effect in solid tumors has not been fully elucidated. In this study, we identified nucleus-localized PFKL (Liver-type phosphofructokinase), as a key regulator of HDACi efficacy and intracellular epigenetic dynamics. Nuclear PFKL directly binds to class I HDACs through interacting with zinc-binding sites, thereby inhibiting HDAC enzymatic activity and promoting intracellular histone acetylation. In addition, the Thr562 residue within PFKL enhances the chelation effect between the zinc-binding group (ZBG) of the HDACi romidepsin and the zinc within the HDACs, further promoting drug efficacy. Based on the mechanism of PFKL facilitates the efficacy of romidepsin, we developed a therapeutic peptide, PFKL-552-572-R8, which significantly enhances the antitumor effect of romidepsin both in vitro and in vivo. Our findings reveal that spatiotemporal regulation confers a moonlight function to PFKL as an endogenous HDAC inhibitor to maintain the stability of epigenetic modifications and highlight PFKL as a promising therapeutic target for enhancing the clinical utility of HDACi in solid tumors.

## Introduction

Cholangiocarcinoma (CCA) is a malignant tumor originating from the biliary tract system, which can be classified into intrahepatic, perihilar, and distal CCA. Chemotherapy with a combination of gemcitabine and platinum is conventional first-line treatment for patients with CCA who are not eligible for surgery. However, patients receiving conventional first-line chemotherapy often have a rapidly worsening performance status, and objective response rate (ORR) of this treatment regimen is generally low.^1^ Although the new standard treatment combining immune checkpoint inhibitors with chemotherapy has improved outcomes in CCA patients, the ORR of this regimen remains below 30%, and it extends the median overall survival of patients by no more than 2 months compared to chemotherapy alone.^2^ Additionally, a significant proportion of CCA patients develop innate or acquired resistance to current therapies, driven by a range of intrinsic and evasive mechanisms that allow cancer cells to bypass inhibitory signals and sustain proliferation. This inevitable resistance poses a formidable obstacle in clinical management. Therefore, the identification of novel and effective treatment strategies for patients with advanced CCA is urgently needed.

Epigenetic modifications, such as acetylation, chromatin remodeling, and DNA methylation, can typically induce heritable changes in cellular phenotypes or gene expression without altering the underlying DNA sequence. These mechanisms play crucial roles in regulating key oncogenic processes such as uncontrolled proliferation, evasion of apoptosis, and metastatic dissemination.^3^ Epigenetic-based therapies represent a promising approach for treating malignant tumors, particularly due to their ability to reverse aberrant gene expression patterns associated with tumor progression.^4^ The application of epigenetic drugs, either alone or in combination with chemotherapy or immunotherapy, has shown convincing effects, including overcoming drug resistance, enhancing the immune response, and boosting anti-tumoral activity.^5,6^ Zinc-dependent histone deacetylases (HDACs) mediate chromatin condensation and transcriptional silencing by removing acetyl groups from histone lysine residues.^7^ HDAC inhibitors can rectify the aberrant acetylation status of histones and trigger apoptosis in cancer cells, including CCA,^8^ breast cancer,^9^ lung cancer,^10^ and T-cell lymphomas,^11^ which highlights HDACs as promising targets in cancer therapy. In clinical settings, HDAC inhibitors have exhibited preferential clinical efficacy and safety profiles primarily within hematologic malignancies.^12,13^ In contrast, single-agent treatment regimens have poor therapeutic effects on nearly all types of solid tumors, which may be attributed to a variety of multifactorial drug resistance mechanisms.^14,15^ These include tumor microenvironment heterogeneity (e.g., hypoxic regions and immune exclusion), compensatory activation of parallel signaling pathways (such as MAPK/ERK or PI3K/AKT), and inadequate intratumoral drug accumulation. The precise molecular mechanisms governing the efficacy of HDAC inhibitors in CCA and other solid tumors remain elusive. In particular, the determinants of sensitivity and resistance are poorly understood, hampering the rational design of combination therapies. Furthermore, current HDAC inhibitors have intrinsic liabilities including inadequate isoform specificity, pharmacokinetic constraints, high toxicity and tumor resistance, which lead to the adverse effects observed in patients undergoing treatment.^16,17^

Glycolysis is a fundamental metabolic pathway conserved across all life forms and is intricately linked to the proliferation, drug resistance, and metastasis of cancer cells.^18^ Many tumors exhibit a metabolic shift known as the Warburg effect, wherein cancer cells preferentially utilize glycolysis for energy production even under normoxic conditions.^19^ As a pivotal rate-limiting enzyme in glycolysis, phosphofructokinase (PFK) catalyzes the irreversible phosphorylation of fructose-6-phosphate (F6P) to generate fructose-1,6-bisphosphate (F1,6BP), a process that is paramount in modulating the pace of glycolytic flux.^20^ In mammals, PFK manifests in three distinct forms: L (liver type, PFKL), M (muscle type, PFKM), or P (platelet type, PFKP).^21^ Most human tissues express all three isoforms, albeit with different levels of expression. Notably, increased expression of PFKL is correlated with increased glycolytic efficiency in aggressive human cancers and has further been linked to poor prognosis and enhanced metastatic potential in several carcinoma types.^22,23^ Recent studies have further elucidated the indispensable role of PFKL in governing phagocytic oxidative burst and cardiac metabolic remodeling.^24,25^ As a gatekeeper of glycolysis, PFKL is localized primarily in either the cytoplasm or the mitochondria.^26^ While the essential metabolic roles of PFKL in modulating metabolic flux and cellular energy homeostasis have been elucidated, its potential specific subcellular localizations and spatiotemporal regulation within the cell remain poorly understood.

In this study, we demonstrated that HDAC inhibitor romidepsin exerts potent cytotoxic effects on CCA cells compared to other epigenetic drugs. Using genome-wide CRISPR-Cas9 screening, we revealed that PFKL deletion specifically confers resistance to HDAC inhibitors in cancer cells. Mechanistically, nucleus-localized PFKL directly interacts with Class I HDACs and inhibits HDAC enzymatic activity. PFKL provides additional interactions at the Thr562 residue and promotes the chelation effect between romidepsin and HDACs, thus enhancing the cytotoxic effect of romidepsin on tumor cells. Based on the mechanism by which PFKL enhances the efficacy of romidepsin, we developed a cell-penetrating therapeutic peptide, PFKL-552-572-R8. This peptide promotes romidepsin-induced HDAC inhibition and enhances antitumor effects both in vitro and in vivo, suggesting its potential application as an adjuvant strategy to overcome HDAC inhibitor resistance in solid tumors.

## Results

### PFKL status determines the therapeutic efficacy of HDAC inhibitors in CCA

**Fig. 1:**
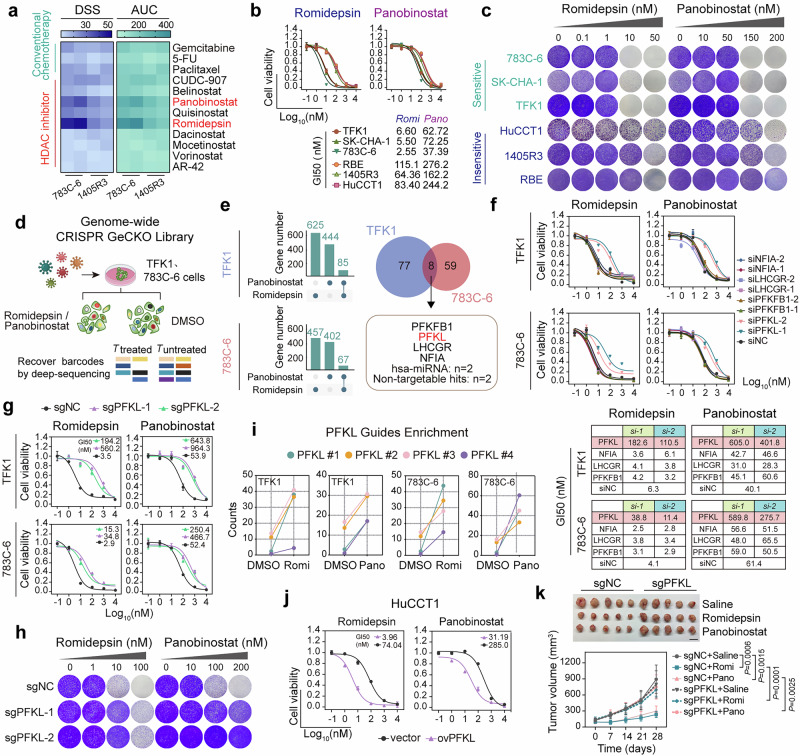
PFKL deficiency triggers HDAC inhibitor tolerance in CCA cells. **a** Drug sensitivity scores (DSS) and areas under the drug-response curve (AUC) of drugs with a DSS score ≥ 10 in the 783C-6 and 1405R3 cell lines. **b** The cell viability of different CCA cell lines following 72 h of treatment with specified concentrations of romidepsin or panobinostat. **c** Colony-formation assay of 783C-6, SK-CHA-1, TFK1, HuCCT1, 1405R3, and RBE cells after 10 days of treatment with specified concentrations of romidepsin or panobinostat. **d** Schematic representation of the genome-wide CRISPR-Cas9 screening process. TFK1 and 783C-6 cells were transduced with a lentiviral genome gRNA library in three independent replicates. gRNA barcodes from the initial time point (T0, day 0) and the subsequent time point (Tu, day 7) were identified via deep sequencing. **e** Eight common hits were identified through CRISPR screening in TFK1 and 783C-6 cells. **f** Assessment of cell viability of TFK1 and 783C-6 cells following 72 h of treatment with romidepsin or panobinostat after interference with NFIA, LHCGR, PFKFB1, or PFKL. **g** Cell viability of TFK1 and 783C-6 cells expressing either a negative control sgRNA (sgNC) or sgRNAs targeting PFKL (sgPFKL-1 and sgPFKL-2) following treatment with specified concentrations of romidepsin or panobinostat for 72 h. **h** The impact of sgRNAs targeting PFKL was evaluated via a colony-formation assay following treatment with specified concentrations of romidepsin or panobinostat for 10 days. **i** Enrichment of sgRNAs targeting PFKL in TFK1 and 783C-6 cells. **j** Cell viability of HuCCT1 cells with vector or PFKL overexpression after 72 h of treatment with the indicated concentrations of romidepsin or panobinostat. **k** Tumor growth rates over time for TFK1-sgNC or TFK1-sgPFKL xenografts with specified treatments. Top panel: Representative tumor images at the end of the treatment. Bottom panel: Growth curves for each group of TFK1-sgNC and TFK1-sgPFKL xenografts (*n* = 5 mice per group, two-way ANOVA). Scale bars, 1 cm. All the statistical data are presented as the means ± SEMs

To probe the inhibitory effects of different epigenetic drugs on CCA cells, we first conducted a high-throughput drug screen utilizing two primary CCA cell lines, 783C-6 and 1405R3. The cells were seeded and incubated with a gradient of concentrations of drugs for 72 h, after which the cell viability was assessed (Supplementary Fig. 1a). The drug library primarily comprises epigenetic drugs, including HDAC inhibitors, DNA methyltransferase inhibitors, and histone demethylase inhibitors, as well as conventional chemotherapies and kinase inhibitors (Supplementary Fig. 1b and Supplementary Table 1). To assess the robustness of the screening assay, we calculated the Z-factor and found that both two cell lines displayed a Z-factor > 0.5, the threshold for a robust difference (Supplementary Fig. 1c). The concentrations resulting in 50% growth inhibition (GI50), area under the curves (AUC) and dose-response curves of each drug were calculated (Supplementary Tables 2 and 3). Based on these data, drug sensitivity scores (DSS) were calculated according to established methods.^27^ For DSS, the cutoff was set at 10, above which a compound was deemed effective (Supplementary Fig. 1d). By intersecting all the selected compounds with DSS values ≥ 10 across two CCA cell lines, we discovered 12 drugs demonstrating broad-spectrum activity against CCA: 9 were HDAC inhibitors and 3 were conventional drugs (Fig. 1a and Supplementary Fig. 1e, f). Among the selected HDAC inhibitors, romidepsin and panobinostat demonstrated optimal cytotoxic effects on CCA cells (Supplementary Fig. 1g, h).

We subsequently assessed the growth inhibitory effects of romidepsin and panobinostat on different CCA cell lines. Notably, CCA cell lines displayed varying sensitivities to the two HDAC inhibitors in both short-term viability assays (Fig. 1b) and long-term clonogenic assays (Fig. 1c). To identify the molecular mechanisms underlying the pharmacological efficacy of HDAC inhibitors in CCA cells, we performed a genome-wide CRISPR-Cas9 screening using two sensitive cell lines 783C-6 and TFK1. We introduced a whole-genome sgRNA library targeting 20,914 genes into CCA cells and treated the cells with romidepsin, panobinostat, or DMSO for 7 days (Fig. 1d). Through positive enrichment of sgRNAs in surviving HDAC inhibitor-treated cells, we identified four protein-coding genes were simultaneously depleted in both TFK1 and 783C-6 cells (Fig. 1e). Subsequent validation using small interfering RNA (siRNA) revealed that only interference with PFKL in CCA cells reduced susceptibility to HDAC inhibitors (Fig. 1f and Supplementary Fig. 2a). Further verification employing sgRNA and shRNA targeting PFKL yielded similar results (Fig. 1g, h and Supplementary Fig. 2b, c). For other HDAC inhibitors, such as belinostat and vorinostat, as well as the cyclic peptide drugs apicidin and HC-Toxin, knockout of PFKL also significantly inhibited drug efficacy (Supplementary Fig. 2d). Additionally, sgRNAs targeting PFKL were significantly enriched in the HDAC inhibitor-treated group (Fig. 1i), suggesting PFKL as a potential regulator of the efficacy of HDAC inhibitors.

We then examined the correlation between PFKL expression and sensitivity to HDAC inhibitors in CCA cells. CCA cells with lower PFKL expression (HuCCT1, 1405R3, and RBE) exhibited reduced sensitivity to HDAC inhibitors, whereas those with higher PFKL expression (TFK1, SK-CHA-1, and 783C-6) presented increased sensitivity, regardless of the HDAC expression level (Fig. 1b and Supplementary Fig. 2e). Overexpression of PFKL in HuCCT1 and 1405R3 cells significantly increased cell sensitivity to HDAC inhibitors (Fig. 1j and Supplementary Fig. 2f, g). Furthermore, deletion of PFKL notably relieved the inhibition of tumor growth caused by HDAC inhibitors in TFK1 xenografts (Fig. 1k and Supplementary Fig. 2h, i), as evidenced by increased expression of the proliferation marker Ki-67 and decreased expression of the apoptotic marker cleaved caspase-3 (Supplementary Fig. 2j, k).

**Fig. 2:**
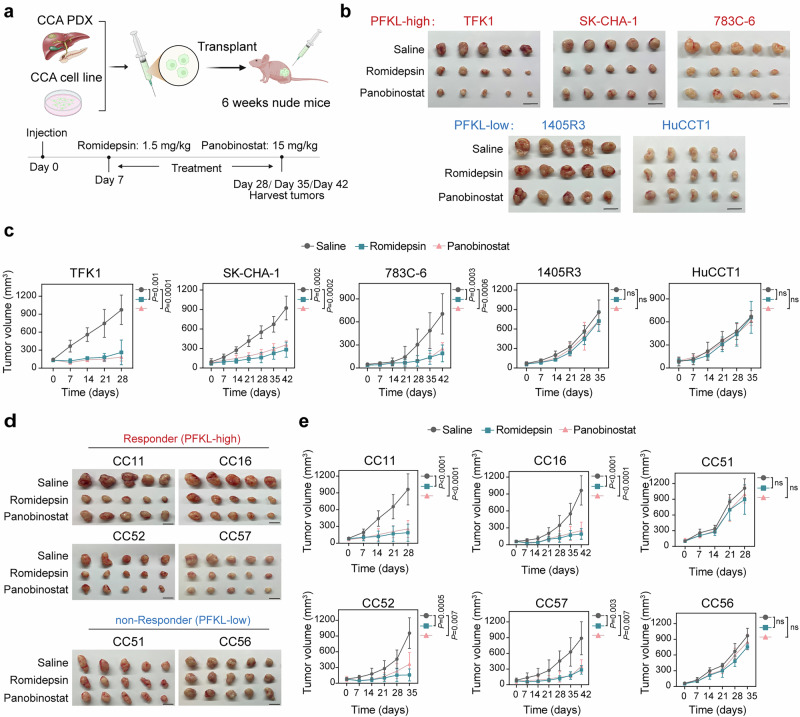
PFKL facilitates the efficacy of HDAC inhibitors in vivo. **a** Schematic diagram of the HDAC inhibitor treatment regimen. **b** Representative tumor images of each group of TFK1, SK-CHA-1, 783C-6, 1405R3, and HuCCT1 xenografts at the end of treatment (*n* = 5 mice per group). Scale bars, 1.5 cm. **c** Growth curves of each group of TFK1, SK-CHA-1, 783C-6, 1405R3, and HuCCT1 xenografts. (*n* = 5 mice per group, two-way ANOVA). **d** Representative images of tumors from various patient-derived xenografts (PDXs) at the end of treatment (*n* = 5 per group). Scale bars, 1 cm. **e** Growth curves of each group across different PDX models (*n* = 5 mice per group, two-way ANOVA). All the statistical data are presented as the means ± SEMs

Next, we investigated the correlation between PFKL expression and the efficacy of HDAC inhibitors in vivo (Fig. 2a). Consistent with the results of the in vitro drug sensitivity assay, romidepsin and panobinostat markedly inhibited tumor growth in mice xenografted with PFKL high-expressed TFK1, SK-CHA-1, and 783C-6 cells. In contrast, HDAC inhibitors exhibited limited efficacy in mice xenografted with PFKL low-expressed HuCCT1 and 1405R3 cells (Fig. 2b, c and Supplementary Fig. 3). Moreover, we established a series of CCA patient-derived xenograft (PDX) models and subsequently treated them with HDAC inhibitors (Supplementary Fig. 4a). Our results demonstrated that romidepsin or panobinostat notably attenuated the growth of CC11, CC16, CC52, and CC57 PDX tumors in vivo, compared to the control group (Fig. 2d, e and Supplementary Fig. 4b, c). However, no significant disparities were observed in CC51 and CC56 tumors, indicating a lack of sensitivity to HDAC inhibitors within these PDX models. Immunohistochemical analyses revealed a substantial reduction of Ki-67 expression in CC11, CC16, CC52, and CC57 tumors following HDAC inhibitor treatment, whereas no significant differences were noted in CC51 and CC56 tumors (Supplementary Fig. 4d, e). Importantly, PFKL exhibited elevated expression levels in responder PDX tumors (CC11, CC16, CC52, and CC57) relative to non-responder tumors (CC51 and CC56), suggesting that PFKL may serve as a biomarker for predicting HDAC inhibitor efficacy (Supplementary Fig. 4f).

### PFKL enhances the efficacy of romidepsin independent of its metabolic function

**Fig. 3:**
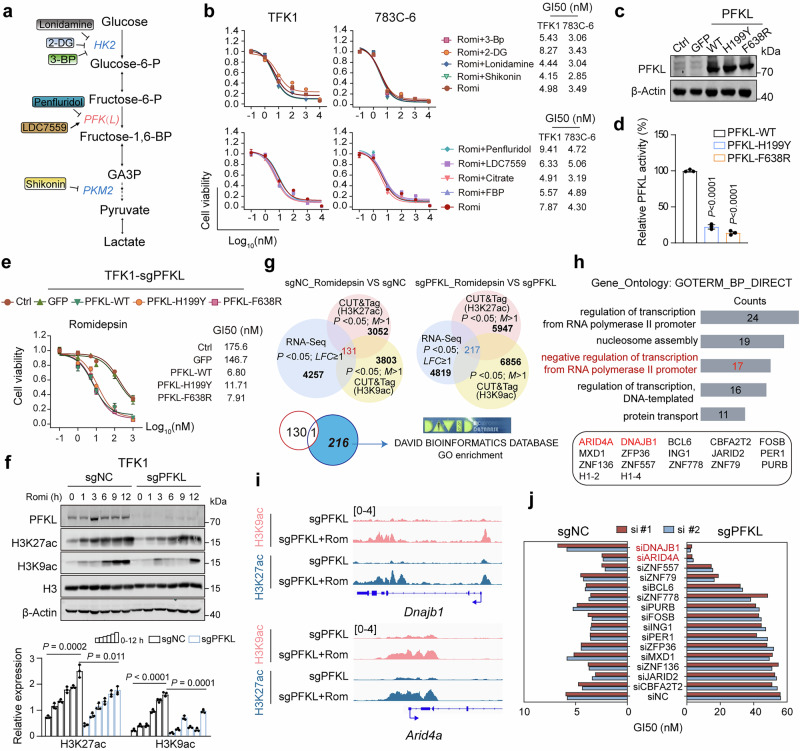
PFKL facilitates the pro-transcriptional effects of romidepsin independent of its metabolic functions. **a** Schematic illustrating the modulation of PFKL enzymatic activity by penfluridol, citrate, FBP and LDC7559 treatments, as well as the inhibition of the glycolytic rate by lonidamine, 2-DG, 3-BP, and shikonin treatments (2-DG, 2-deoxy-D-glucose; 3-BP, bromopyruvic acid). **b** The cell viability of TFK1 and 783C-6 cells after 72 h of treatment with specified concentrations of romidepsin combined with glycolysis inhibitors and the PFKL enzymatic activity modifier. **c** Immunoblot analyses of the expression of PFKL in PFKL-depleted TFK1 cells transfected with the GFP, PFKL-WT, PFKL-H199Y, and PFKL-F638R plasmids. **d** Enzymatic activity of purified wild-type PFKL (PFKL-WT), and PFKL mutants H199Y and F638R (*n* = 3 biological replicates, one-way ANOVA). **e** Cell viability of TFK1-sgPFKL cells treated with specified concentrations of romidepsin for 72 h post-transfection with PFKL-WT, PFKL-H199Y, or PFKL-638R. **f** Immunoblot analyses of the expression of H3K9ac and H3K27ac in TFK1 cells expressing either a negative control sgRNA (sgNC) or sgRNA targeting PFKL (sgPFKL) following treatment with specified concentrations of romidepsin for different durations (*n* = 3 biological replicates, two-way ANOVA). **g** CUT&Tag combined with RNA-Seq data for bioinformatics analysis via the DAVID database. **h** GO enrichment analysis of the pathways specifically enriched in PFKL-deficient cells response to romidepsin. **i** Normalized read densities for H3K9ac and H3K27ac at the *Dnajb1* and *Arid4a* genes. **j** Histogram showing the GI50 values of romidepsin TFK1-sgNC and TFK1-sgPFKL cells following interference with candidate genes. All the statistical data are presented as the means ± SEMs

Compared to panobinostat, romidepsin exhibited superior cytotoxic effects in both cellular and animal models. Consequently, we focused on romidepsin to investigate the regulatory role of PFKL in HDAC inhibitor efficacy. Considering that PFKL plays a pivotal role in the glycolytic pathway, we specifically investigated its metabolic function in modulating the efficacy of romidepsin (Fig. 3a). Despite the prominent reduction in the glycolysis rate due to PFKL deletion (Supplementary Fig. 5a), treatment with glycolytic inhibitors did not elicit significant changes in the sensitivity of TFK1 cells to romidepsin (Fig. 3b and Supplementary Fig. 5b). Moreover, we found that manipulating PFKL enzymatic activity with inhibitors (citrate and penfluridol^28^) or agonists (FBP and LDC7559^25^) did not significantly alter cell sensitivity to romidepsin (Fig. 3b and Supplementary Fig. 5c). We subsequently generated enzymatically inactive PFKL-F638R and PFKL-H199Y mutants as reported previously^29^ and transfected them into PFKL-deleted TFK1 cells (Fig. 3c, d), followed by the assessment of cell sensitivity to romidepsin. Notably, both wild-type and enzymatically inactive PFKL restored the sensitivity of PFKL-deleted TFK1 cells to romidepsin (Fig. 3e). Additionally, interference with PFKP or PFKM did not affect the sensitivity of TFK1 cells to romidepsin (Supplementary Fig. 5d, e), underscoring the specificity of PFKL in modulating the efficacy of romidepsin.

Given that romidepsin inhibits HDAC enzymatic activity following cellular uptake and intracellular reduction,^30,31^ we also explored the potential impact of PFKL on these crucial processes. The results showed that differential expression of PFKL did not affect the cellular uptake of romidepsin or GSH levels, which are crucial for the reduction of romidepsin (Supplementary Fig. 5f–h). These data suggest that PFKL enhances the efficacy of romidepsin independent of its metabolic function.

### PFKL promotes romidepsin-induced transcriptional activation and inhibits HDAC enzymatic activity

Romidepsin inhibits HDAC enzymatic activity by chelating Zn^2+^ within intracellular HDACs, thereby increasing histone acetylation, promoting the transcription of tumor suppressor genes, and inducing growth inhibition in tumor cells.^31^ Consistent with previous reports, we observed a gradual increase in acetylation at the H3K9 and K27 histone sites in TFK1 cells with prolonged romidepsin treatment, whereas PFKL deletion restricted this induction (Fig. 3f). Next, we performed enrichment analysis on differential peaks via CUT&Tag from the comparative groups sgPFKL_romidepsin vs. sgNC_romidepsin (H3K9/K27ac) (Supplementary Tables 4 and 5). Annotation of differential peaks in promoter regions (<3 kb) identified 3387 genes with upregulated expression and 1015 genes with downregulated expression (Supplementary Fig. 6a). Gene Ontology (GO) enrichment analysis of these differentially expressed genes revealed that PFKL knockout significantly enhanced the cellular damage response and DNA repair under romidepsin treatment relative to sgNC controls, suggesting that PFKL depletion promotes romidepsin resistance (Supplementary Fig. 6a). To elucidate the regulatory role of PFKL in gene expression upon romidepsin treatment, we next integrated CUT&Tag with RNA-seq data and identified 216 genes whose expression specifically increased in PFKL-deleted TFK1 cells following romidepsin treatment (Fig. 3g). Subsequent GO enrichment analysis via the DAVID database revealed that these genes are associated primarily with biological processes such as transcriptional regulation and nucleosome assembly (Fig. 3h). Notably, enrichment analysis highlighted a significant upregulation of the negative regulation of transcription from RNA polymerase II promoter signaling pathway, suggesting a potential antagonistic effect of PFKL deletion on the pro-transcriptional activity of romidepsin (Fig. 3h). Validation of H3K9ac and H3K27ac peaks at the promoters of selected genes showed marked increases in PFKL-deleted TFK1 cells treated with romidepsin (Fig. 3i and Supplementary Fig. 6b). We designed siRNAs targeting 15 selected genes to assess their impact on the efficacy of romidepsin in PFKL-deleted TFK1 cells (Supplementary Fig. 6c). Specifically, interference with DNAJB1 or ARID4A significantly restored the sensitivity of TFK1 cells to romidepsin (Fig. 3j and Supplementary Fig. 6d, e), suggesting a pivotal role of DNAJB1 or ARID4A in PFKL enhancing the efficacy of romidepsin.

**Fig. 4:**
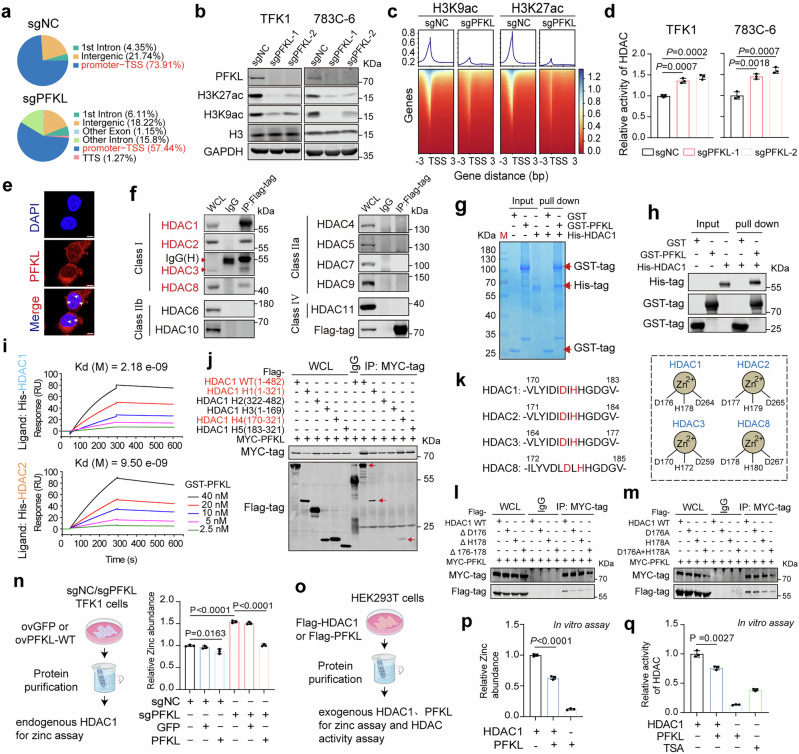
PFKL directly interacts with class I HDACs to modulate cellular epigenetic states. **a** Pie chart illustrating the distribution of annotated genomic regions in TFK1 cells expressing either sgNC or sgPFKL. **b** Immunoblot analysis of H3K9ac and H3K27ac expression in TFK1 and 783C-6 cells expressing either sgNC or sgPFKL. **c** Heatmap illustrating the genomic occupancy of H3K9ac and H3K27ac within regions flanking transcription start sites (±3 kb) in TFK1-sgNC and -sgPFKL cells. Genes, depicted as rows, are sorted in descending order by signal strength. **d** Detection of HDAC enzymatic activity in TFK1 and 783C-6 cells expressing sgNC or sgPFKL (*n* = 3 biological replicates, one-way ANOVA). **e** IF images of PFKL and DAPI staining in TFK1 cells. Scale bars, 8 μm. **f** Coimmunoprecipitation (co-IP) showing the interaction between zinc-dependent HDACs and Flag-tagged PFKL in TFK1 cells. **g**, **h** Coomassie brilliant blue staining and immunoblot analysis of a GST pull-down assay illustrating the interaction between GST-PFKL and His-HDAC1. Red arrows indicate corresponding bands. **i** The binding affinity detected by surface plasmon resonance (SPR) between GST-PFKL and His-HDAC1 or His-HDAC2. **j** Co-IP showing the interactions between MYC-tagged PFKL and Flag-tagged full-length or truncated HDAC1 in HEK293T cells. **k** Amino acid sequence of the PFKL-binding region in Class I HDACs. Zinc-binding residues are color-marked. Schematic diagram of the zinc-binding residues in Class I HDACs. **l**, **m** Co-IP showing the interactions between MYC-tagged PFKL and Flag-tagged full-length or zinc-binding residue mutant HDAC1 in HEK293T cells. **n** Zinc abundance in HDAC1 purified from TFK1 cells expressing sgNC, sgPFKL, GFP, or wild-type (WT) PFKL (*n* = 3 biological replicates, one-way ANOVA). **o**–**q** In vitro zinc abundance and enzymatic activity of purified Flag-tagged human HDAC1 protein from HEK-293T cells following incubation with purified PFKL (*n* = 3 biological replicates, one-way ANOVA). All the statistical data are presented as the means ± SEMs

The above results confirmed that PFKL deletion reduced the pro-transcriptional effects of romidepsin without affecting drug uptake or intracellular reduction, suggesting that PFKL directly regulates the downstream effects of romidepsin, especially histone acetylation and gene transcription. To explore this hypothesis, we initially conducted assay for transposase-accessible chromatin with sequencing (ATAC-seq) and observed a significant reduction in the proportion of promoter-transcription start site (TSS) regions in PFKL-depleted TFK1 cells (Fig. 4a). Acetylation of histone H3K9 and H3K27 sites is closely associated with the positive regulation of gene transcription.^32^ Notably, PFKL deletion led to a significant decrease in the acetylation of H3K9 and H3K27 sites, while overexpression of PFKL caused the opposite effect (Fig. 4b and Supplementary Fig. 7a). Consistent with our immunoblot assay findings, we also observed diminished H3K9ac and H3K27ac peaks near TSS regions in PFKL-depleted cells using CUT&Tag (Fig. 4c).

Given that alterations in cellular lactate and lactylation modifications can regulate the acetylation status of H3K9 and K27 sites,^33^ we first investigated whether the reduced lactate abundance caused by PFKL deletion directly affects the expression of acetyl-H3K9 and acetyl-H3K27. Depletion of PFKL resulted in reduced levels of global lysine lactylation (pan-Kla), which may be associated with decreased intracellular lactate content (Supplementary Figs. 5a and 7b). Notably, transfection with either wild-type or enzymatically inactive PFKL in PFKL-depleted TFK1 cells significantly promoted the acetylation of H3K9 and K27, suggesting that the altered lactate content due to PFKL depletion may not be the key factor affecting histone acetylation (Supplementary Fig. 7c). Histone acetylation is dynamically regulated by histone acetyltransferases (HATs) and HDACs, which catalyze the addition and removal of acetyl groups from lysine residues on histones, respectively.^34^ PFKL deficiency did not influence HAT enzymatic activity, suggesting that PFKL-mediated histone acetylation is independent of HATs (Supplementary Fig. 7d). In contrast, HDAC enzymatic activity was significantly enhanced upon PFKL deletion in TFK1 or 783C-6 cells and reduced upon PFKL overexpression in HuCCT1 cells (Fig. 4d and Supplementary Fig. 7e). These findings collectively indicate that PFKL regulates histone acetylation through the suppression of HDAC enzymatic activity.

### PFKL directly interacts with class I HDACs to modulate epigenetic states

We further investigated how PFKL suppresses HDAC enzymatic activity. Previous studies have indicated that phosphorylation at serine sites of HDACs affects their enzymatic activity.^35,36^ Using HDAC1 as a model, we investigated whether PFKL influences the phosphorylation of HDACs. Our results demonstrated that PFKL did not affect the expression of p-HDAC1 (Ser421, Ser423) (Supplementary Fig. 8a, b). PFKL is generally localized in the cytoplasm and mitochondria to regulate glycolytic metabolism. Given that class I HDACs, which are the main targets of romidepsin, are located mainly in the nucleus, we speculate that PFKL may regulate HDAC enzymatic activity through its special intracellular localization. We analyzed the subcellular localization of PFKL via immunofluorescence assays. In addition to detecting the predominant presence of PFKL in the cytoplasm, we also observed its localization in the nucleus (Fig. 4e). We subsequently examined whether PFKL directly interacted with HDACs via coimmunoprecipitation (co-IP) and immunofluorescence assays. Intriguingly, PFKL was found to bind predominantly to class I HDACs (HDAC1, HDAC2, HDAC3, and HDAC8) among the zinc-dependent HDACs (Fig. 4f and Supplementary Fig. 8c). The interactions between endogenous PFKL and class I HDACs were further validated in TFK1 whole-cell lysates and nuclear lysates (Supplementary Fig. 8d, e). Additionally, GST pull-down and surface plasmon resonance (SPR) techniques confirmed the direct interaction between PFKL and class I HDACs in cell-free assays (Fig. 4g–i and Supplementary Fig. 8f–m). Furthermore, mass spectrometry analysis of the HDAC1 immunoprecipitates confirmed the presence of several reported HDAC complexes (such as MTA1 and sin3A) and identified PFKL as a potential interacting protein (Supplementary Fig. 8n and Supplementary Table 6). Mass spectrometry analysis of PFKL immunoprecipitates likewise confirmed the HDAC1‒PFKL interaction (Supplementary Fig. 8o and Supplementary Table 6). Co-IP results for the three distinct PFK isoforms demonstrated that PFKL does not bind to PFKP and only weakly interacts with PFKM, suggesting that PFKL is more likely to form homologous homotetramers (PFKL₄) than heterotetramers^37^ (PFKL/PFKM) in TFK cells (Supplementary Fig. 8p). Notably, we observed that PFKL, but not PFKM or PFKP, specifically interacts with HDAC1 (Supplementary Fig. 8q, r). Collectively, these findings suggest that PFKL directly binds to class I HDACs, consequently inhibiting their enzymatic activity.

Given the high conservation of amino acid sequences among several class I HDAC proteins, we selected HDAC1 to verify the key domain involved in its interaction with PFKL. To delineate the domain responsible for the interaction between HDAC1 and PFKL, HEK293T cells were transfected with several truncated HDAC1 constructs alongside PFKL plasmids for co-IP assays. Our findings revealed that only the H1 region (aa 1-321) and H4 region (aa 170-321) of HDAC1, but not the H5 region (aa 183-321), interacted with PFKL. This finding suggests that the amino acid residues of the 170-183 region in HDAC1 mediate its binding to PFKL (Fig. 4j and Supplementary Fig. 9a, b). We also confirmed that the C-terminal regulator region of PFKL (aa 401-780) is crucial for its binding to HDAC1 (Supplementary Fig. 9c, d). Furthermore, to explore the mechanism of PFKL nuclear import, we conducted bioinformatic analyses and identified several candidate nuclear localization signals (NLSs) within PFKL, including residues 712–715 (LKKK, a classical basic motif), 624–628 (VLRNE), and 563–566 (KRRV, a single basic cluster). To test these motifs functionally, we generated three Myc-tagged PFKL deletion constructs (Δ712–715, Δ624–628, and Δ563–566) and expressed them in PFKL-knockout TFK1 cells. We then assessed the subcellular distribution of these proteins via both nuclear‒cytoplasmic fractionation and immunofluorescence assays. However, none of these deletions led to a significant reduction in nuclear localization, suggesting that the tested regions may not be solely responsible for nuclear targeting (Supplementary Fig. 9e, f). This finding raises the possibility that PFKL may harbor additional, noncanonical NLS sequences or that nuclear import may be mediated through interaction with nuclear transport proteins (e.g., by binding to HDACs or other carriers).

Since the 170-183 region is highly conserved across the four Class I HDAC protein sequences, we defined this region as the “PKL-binding region” here (Fig. 4k). Notably, two of the three Zn-binding residues in HDAC1 (D176 and H178) are located within the PFKL-binding region, and these residues are also conserved among Class I HDACs (Fig. 4k). Specifically, deletion or mutation of D176 or H178 diminished the interaction between HDAC1 and PFKL (Fig. 4l, m). Given the pivotal role of Zn-binding residues in regulating zinc homeostasis and the enzyme activities of zinc-dependent HDACs, we examined the zinc abundance of HDAC1 purified from control or PFKL-depleted TFK1 cells. The results showed that PFKL depletion caused a substantial increase in the zinc content of HDAC1, which could be reversed by exogenous PFKL expression (Fig. 4n). By purifying the class I HDAC and PFKL proteins in vitro, we also demonstrated that the addition of PFKL protein markedly reduced the zinc abundance in class I HDAC protein, consequently repressing its enzymatic activity (Fig. 4o–q and Supplementary Fig. 10a). In addition, class I HDACs constitute the catalytic subunit of a variety of transcriptional regulatory complexes. Class I HDACs (except HDAC8) are recruited into multi-subunit complexes by direct binding to one of at least 17 corepressor proteins.^38^ Our results indicated that PFKL depletion does not seem to have a significant effect on the interaction between class I HDACs and complex subunits, suggesting that PFKL may regulate HDAC enzymatic activity by directly binding to HDACs rather than indirectly affecting the interaction between multiple complex subunits and the HDAC protein (Supplementary Fig. 10b–e).

Given that nuclear PFKL directly interacts with class I HDACs and inhibits their enzymatic activity and that a variety of HDAC inhibitors (such as romidepsin) chelate with zinc within HDACs in the nucleus, we also explored the ability of nuclear PFKL to predict the efficacy of HDAC inhibitors. Consistent with previous results, the expression of PFKL in the nucleus was positively correlated with the total amount of PFKL in CCA cell lines (Supplementary Fig. 11a, b). Moreover, PDX tumors (CC11, CC16, CC52, and CC57) with increased expression of nuclear PFKL were more sensitive to HDAC inhibitor treatment (Fig. 2e and Supplementary Fig. 11c). Additionally, a continuous duration of romidepsin treatment had no significant effect on the overall expression level of PFKL or its subcellular localization (Supplementary Fig. 11d, e).

### PFKL facilitates the chelation effect between romidepsin and zinc within HDAC1

**Fig. 5:**
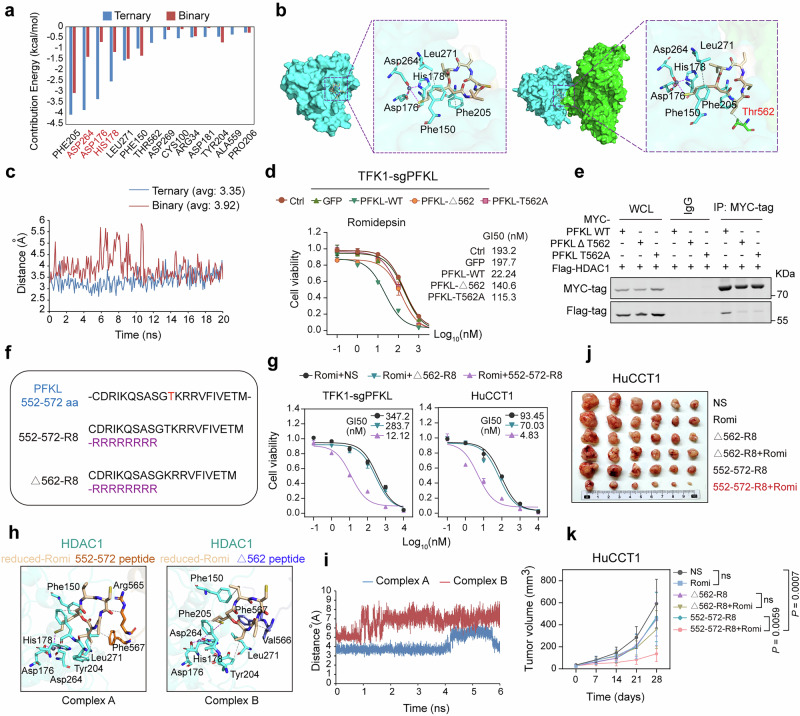
The PFKL-552-572-R8 peptide increases the efficacy of romidepsin in vitro and in vivo. **a** Histogram showing the energy contribution of crucial residues in binary (HDAC1 and reduced-romidepsin) and ternary (HDAC1, reduced-romidepsin, and PFKL) complexes. **b** Molecular dynamics clustering analysis of binding modes in binary and ternary complexes. HDAC1 is represented by a cyan surface, PFKL is depicted with a green surface, and key residues are displayed as cyan and green sticks, respectively. The HDAC inhibitor romidepsin is shown as wheat-colored sticks, and zinc is represented as a gray sphere. The purple dashed lines denote coordination bonds, and the gray dashed lines indicate hydrophobic interactions. **c** Line chart showing the distances between the zinc and sulfur atoms of romidepsin in binary and ternary complexes. Binary complexes are represented in red, whereas ternary complexes are depicted in blue. **d** Cell viability of TFK1-sgPFKL cells treated with specified concentrations of romidepsin for 72 h post-transfection with PFKL-WT, PFKL-∆562, or PFKL-T562A. **e** Co-IP showing the interactions between Flag-tagged HDAC1 and Myc-tagged full-length or Thr562 mutant PFKL in HEK293T cells. **f** Schematics of the amino acid sequences of the peptides. **g** Cell viability of TFK1-sgPFKL and HuCCT1 cells treated with specified concentrations of romidepsin for 72 h combined with normal saline (NS), 552-572-R8, or ∆562-R8. **h** Molecular dynamics clustering analysis of binding modes in HDAC1-reduced romidepsin-peptide complexes. HDAC1 is shown as a blue-green (cyan) cartoon (cartoon), 552572-R8 and ∆562-R8 are shown as orange (orange) and dark blue-gray (slate) cartoons, respectively. Romidepsin is shown as a wheat (wheat) stick, and zinc ions are shown as a gray (gray) sphere (sphere); the key residues are presented as sticks (stick). The gray dashed line indicates hydrophobic interactions, and the magenta (magenta) dashed line indicates coordination. **i** Line chart showing the distances between the zinc and sulfur atoms of romidepsin in the two complexes. Complex A is represented in blue, whereas complex B is depicted in red. **j** Representative tumor images of each group of HuCCT1 xenografts at the end of treatment (*n* = 6 mice per group). **k** Growth curves of each group of HuCCT1 xenografts (*n* = 6 mice per group, two-way ANOVA). All the statistical data are presented as the means ± SEMs

To gain further insight into the structural basis by which PFKL regulates the efficacy of romidepsin, we employed DOCK 6.9 and HDOCK SERVER to conduct molecular docking analysis of the ternary complex comprising HDAC1, PFKL, and reduced-romidepsin. Our analysis revealed a substantial similarity in the conformations of several ternary complexes. Specifically, reduced-romidepsin binds between HDAC1 and PFKL, with its linker and zinc-binding group (ZBG) inserting into the Zn-catalyzed channel of HDAC1, thereby coordinating with zinc (Supplementary Fig. 12a). Upon removing PFKL from the stabilized ternary complex, we obtained a PFKL-free binary complex. Analysis of the root mean squared deviation (RMSD) of both binary and ternary complexes revealed that both conformations could achieve stability shortly after molecular dynamics simulations (Supplementary Fig. 12b, c). Additionally, we observed that the binding free energy of the ternary complex (Δ Gtotal = −28.1249 kcal/mol) was lower than that of the binary complex (Δ Gtotal = −20.9573 kcal/mol), indicating that reduced-romidepsin can bind more stably to the ternary complex containing PFKL (Supplementary Fig. 12d). Subsequent energy decomposition calculations of key residues involved in the binding process revealed that the presence of PFKL could significantly influence the number of Zn-binding residues (Fig. 5a). Further analysis of representative conformations showed that reduced-romidepsin in the ternary complex not only interacts with key residues of HDAC1 but also engages with the Thr562 residue of PFKL, thereby increasing binding stability (Fig. 5b). Moreover, we observed that the Cap group of reduced-romidepsin was more deeply buried within the protein, resulting in a reduction in the solvent access surface area (SASA). Importantly, the ZBG of reduced-romidepsin in the ternary complex is liganded closer to the zinc in HDAC1 compared to the binary complex, suggesting a superior inhibitory effect (Fig. 5c). Considering that the intracellular content and working concentration of romidepsin have potential effects on ternary complex formation and interactions between PFKL and class I HDACs, we subsequently explored the effects of cellular GI50-based doses and overdoses of romidepsin on the interactions between PFKL and HDACs, as well as changes in the intracellular content of romidepsin within the duration of drug treatment. The results showed that romidepsin did not significantly affect the interactions between HDACs and PFKL even at doses exceeding the GI50 values (Supplementary Fig. 12e). In addition, after treating cells with romidepsin for 12 h, the drug was removed, and the changes in intracellular drug content over time were subsequently detected. Within 24 h of drug withdrawal, there was no significant change in the intracellular romidepsin content, with only a slight decrease observed after 48 h (Supplementary Fig. 12f). This is sufficient to induce the formation of ternary complexes and downstream drug effects (such as the induction of tumor cell apoptosis).

### PFKL-derived peptide 552-572-R8 potentiates romidepsin efficacy and expands its application in solid tumors

Considering that Thr562 in PFKL plays a critical role in maintaining the affinity of romidepsin targeting HDAC, we further validated the influence of Thr562 on drug efficacy by transfecting TFK1-sgPFKL cells with Thr562 deletion or mutation PFKL plasmids. As expected, only wild-type PFKL significantly enhanced the efficacy of romidepsin, while PFKL with a deletion or mutation at Thr562 was insufficient to induce notable changes in cellular drug sensitivity (Fig. 5d and Supplementary Fig. 13a). Additionally, we also observed that the deletion or mutation of Thr562 in PFKL significantly reduced its binding to HDAC1 (Fig. 5e). These results underscore the critical role of Thr562 in PFKL for maintaining protein-protein interactions and drug efficacy.

Next, we synthesized two peptides centered around the PFKL Thr562 residue, PFKL-552-572-R8 and 552-572-∆562-R8 (∆562-R8), each containing a C-terminal fusion of eight arginine residues (R8) to facilitate cellular uptake and nuclear targeting (Fig. 5f). The peptides exhibited good cellular permeability and low cytotoxicity (Supplementary Fig. 13b, c). To assess the effects of the two peptides on the efficacy of romidepsin, TFK1-sgPFKL cells and HuCCT1 cells were pretreated with the peptides for 12 h, followed by the addition of romidepsin for 72 h to detect cell viability. Remarkably, treatment with the 552-572-R8 peptide, but not the ∆562-R8 peptide, significantly increased cell sensitivity to romidepsin (Fig. 5g). Although treatment with the peptides alone does not alter the histone acetylation levels in cells, the 552-572-R8 peptide significantly enhances the intracellular acetylation levels under romidepsin treatment (Supplementary Fig. 13d, e).

To further understand the structural basis by which peptides affect the efficacy of romidepsin, we performed molecular docking of the peptide, HDAC1, and reduced-romidepsin to obtain an appropriate ternary complex conformation (Supplementary Fig. 13f). We examined the salt-bridge interactions between the peptide and the HDAC1 protein. The results showed that the 552-572 peptide formed three stable salt bridges with residues on the upper and lower interfaces of HDAC1 (Supplementary Fig. 13g). However, in the peptide lacking Thr562, only one salt bridge was detected, with a lower occupancy than that of the 552-572 peptide (Supplementary Fig. 13g and Supplementary Table 7). These findings further indicate that the deletion of Thr562 induces conformational changes in the peptide, reducing polar interactions between the peptide and the HDAC1 protein. Finally, through clustering analysis of molecular dynamics trajectories, we found that the conformational changes caused by the deletion of Thr562 in the peptide significantly altered the residues responsible for the hydrophobic interaction between the peptide and reduced-romidepsin, and this change further increased the coordination bond distance between the ZBG of reduced-romidepsin and zinc within HDAC1, leading to a reduced chelation effect (Fig. 5h, i).

We next sought to evaluate the in vivo effects of the two peptides. HuCCT1 cells, which exhibit low PFKL expression, and their corresponding xenografts displayed limited sensitivity to romidepsin treatment. Notably, administration of the 552-572-R8 peptide, but not the Δ562-R8 peptide, markedly suppressed HuCCT1 tumor growth when it was combined with romidepsin (Fig. 5j, k and Supplementary Fig. 13h–j), highlighting its potential to enhance the therapeutic efficacy of romidepsin in solid tumors.

We subsequently investigated the efficacy of several HDAC inhibitors across a broad spectrum of cancer cell lines through integrating data from the Genomics of Drug Sensitivity in Cancer (GDSC) database. The results indicated that hematological tumor cell lines appeared to be more sensitive to romidepsin treatment, which aligns with the clinical outcomes of HDAC inhibition (Supplementary Fig. 14a). Additionally, we analyzed the drug sensitivity data of another class I HDAC inhibitor, entinostat, and obtained similar results (Supplementary Fig. 14b). Considering the superior efficacy of HDAC inhibitors in hematologic tumors, we conducted a comprehensive investigation into the mutation status and mRNA expression of PFKL in various tumor cells sourced from the Cancer Cell Line Encyclopedia (CCLE). Our analysis revealed a consistent occurrence of amplified mutations and elevated expression of PFKL in hematological tumors, including B-cell lymphoma and myeloproliferative neoplasms, further supporting the notion that PFKL enhances the effectiveness of HDAC inhibitors (Supplementary Fig. 14c, d).

**Fig. 6:**
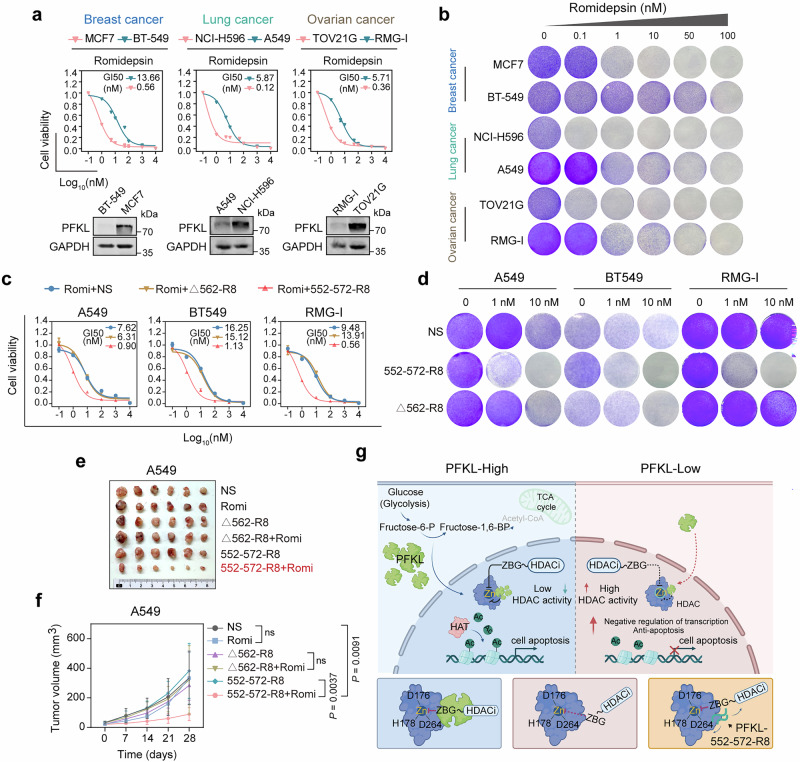
The PFKL-552-572-R8 peptide broadens the application of romidepsin in various solid tumors. **a** Cell viability of different solid tumor cell lines following treatment with specified concentrations of romidepsin for 72 h. **b** Colony-formation assay of MCF7, BT-549, NCI-H596, A549, TOV21G, and RMG-1 cells following treatment with specified concentrations of romidepsin for 10 days. **c** Cell viability of romidepsin-resistant cells following treatment with specified concentrations of romidepsin combined with NS, 552-572-R8, or ∆562-R8 for 72 h. **d** Colony-formation assay of romidepsin-resistant cells following treatment with specified concentrations of romidepsin combined with normal saline (NS), 552-572-R8, or ∆562-R8 for 10 days. **e** Representative tumor images of each group of A549 xenografts at the end of treatment (*n* = 6 mice per group). **f** Growth curves of each group of A549 xenografts (*n* = 6 mice per group, two-way ANOVA). **g** Schematic representation of PFKL regulation of epigenetic states and the efficacy of HDAC inhibitors in cancer. All the statistical data are presented as the means ± SEMs. Image created with BioRender.com

To extend the scope of our findings, we selected six solid cancer cell lines exhibiting varying levels of PFKL expression. Consistently, cells with increased expression of total or nuclear PFKL displayed increased sensitivity to romidepsin treatment across breast, lung, and ovarian cancer cell lines (Fig. 6a, b and Supplementary Fig. 15a, b). Consistent with the findings in CCA cells, knockdown of PFKL conferred resistance to romidepsin, while overexpression promoted the drug sensitivity (Supplementary Fig. 15c–h), underscoring the significant influence of PFKL expression on romidepsin sensitivity in other solid tumor cells.

For solid tumor cell lines resistant to romidepsin treatment, the sensitizing efficacy of the 552-572-R8 peptide was confirmed in both short-term cell viability assays and long-term clonogenic assays (Fig. 6c, d). Importantly, in vivo drug sensitivity experiments with lung cancer A549 xenografts demonstrated that the combination of the 552-572-R8 peptide with romidepsin treatment significantly inhibited tumor growth, suggesting the potential benefits of the 552-572-R8 peptide in enhancing the drug efficacy of romidepsin (Fig. 6e, f and Supplementary Fig. 16).

## Discussion

Following DNA methyltransferase (DNMT), HDAC inhibition emerges as a promising avenue in epigenetic drug discovery.^39,40^ Since the first report of an effective zinc-dependent HDAC inhibitor over three decades ago, five HDAC inhibitors have received regulatory approval for cancer chemotherapy, with numerous others in clinical development across oncology and other therapeutic indications.^13,41,42^ While HDAC inhibitors have demonstrated efficacy with a tolerable safety profile primarily in hematological cancers, challenges, including low response rates and drug resistance, persist in solid tumor treatment. Our data provide compelling evidence highlighting PFKL as a pivotal determinant governing the efficacy of HDAC inhibitors. Notably, the regulatory role of PFKL in the sensitivity of cells to HDAC inhibitors remains independent of its metabolic function. PFKL with nuclear localization not only interacts with class I HDACs but also establishes a stable association with romidepsin, enhancing the targeted binding affinity of romidepsin to zinc within HDACs. PFKL deletion results in an extended chelation distance between the ZBG group of romidepsin and zinc, consequently diminishing the efficacy of romidepsin. On the basis of this mechanism, we identified the 552-572-R8 peptide, which can promote chelation effect between romidepsin and zinc within class I HDACs, subsequently enhancing antitumor efficacy of romidepsin both in vitro and in vivo. Furthermore, this study identified DNAJB1 and ARID4A, genes implicated in transcriptional regulation and antiapoptotic processes,^43,44^ as downstream targets of PFKL regulate the efficacy of romidepsin. Enhanced negative regulation of transcription and antiapoptotic activity due to PFKL deletion collectively contribute to cellular resistance to HDAC inhibitors (Fig. 6g). Overall, these findings highlight PFKL as a promising biomarker for predicting the efficacy of HDAC inhibitors, offering new insights to broaden the clinical utility of HDAC inhibitors in solid tumors.

Aerobic glycolysis is widely recognized as a crucial contributor to cancer pathogenesis, with PFK-1, particularly its isoform PFKL, exerting a profound influence on glycolytic flux during oncogenic progression.^45,46^ As a key rate-limiting enzyme, PFKL orchestrates the upregulation of glycolysis and promotes the proliferation of tumor cells. Thus, targeting PFKL to attenuate glycolysis has potent antitumor effects.^22^ Moreover, PFKL was shown to play a central role in the synergistic control of glycolytic flux, mitochondrial integrity, and lipid homeostasis.^47^ However, our study revealed a previously unrecognized role of PFKL in epigenetic regulation within cancer cells. Although PFKL is highly expressed in the liver, its canonical role is confined to the cytoplasm, where it forms active tetramers to facilitate glycolysis. It is plausible that in normal hepatic physiology, the overwhelming abundance and polymerization of PFKL in the cytoplasm minimizes its availability for nuclear translocation and interaction with HDAC1. In contrast, our study was conducted in cancer cells (especially liver cancer), a setting of cellular transformation and altered metabolic regulation. Oncogenic reprogramming is well known to disrupt normal cellular compartmentalization and promote the noncanonical functions of metabolic enzymes. Thus, the pathological environment of cancer might be a critical prerequisite for the nuclear localization of PFKL and its subsequent engagement with HDAC1 to regulate gene expression. This context dependency likely explains why such an interaction was not previously identified in studies focusing on normal tissues or other cell types.

Zinc, an essential trace element, serves as a critical structural and catalytic cofactor for numerous proteins, and its dysregulation has been implicated in various diseases, including cancer.^48^ The intricate mechanisms governing intracellular zinc metabolism have been elucidated in prior investigations, encompassing processes such as zinc uptake, intracellular transport, and efflux.^49^ A recent study has unveiled the existence of a family of Zn metallochaperones and identified the pivotal role of Zn-regulated GTPase metalloprotein activator (ZNG1) for intracellular zinc trafficking.^50^ Here, we demonstrate that PFKL significantly diminishes the zinc levels within class I HDACs. It remains uncertain whether PFKL functions akin to the metallochaperone ZNG1, or whether it participates in a process termed “zinc stability” intrinsic to individual proteins, wherein zinc homeostasis within zinc-dependent proteins is precisely governed by additional molecules. PFKL likely serves as a key regulatory protein in zinc stabilization by precisely modulating zinc levels within HDACs through its interaction with class I HDACs. Given the critical role of zinc-binding residues D176 and H178 in HDAC1 for PFKL binding, along with the impact of PFKL on zinc-binding residues, this hypothesis appears plausible. Moreover, these findings prompt further exploration into the specific mechanism by which PFKL regulates zinc levels within class I HDACs.

The stabilization of the Zn domain is essential for preserving HDAC enzymatic activity.^51,52^ Given the ability of PFKL to interact with the zinc-binding sites in class I HDACs, it is conceivable that HDAC inhibitors designed to directly target the Zn domain may disrupt the interaction between PFKL and the zinc-binding sites. Meanwhile, it is imperative to thoroughly assess the potential alterations and perturbations in gene transcription resulting from direct targeting of the Zn domain during the pursuit of novel HDAC inhibitors.

Class I HDACs possess analogous Zn domains and can be incorporated into different complexes, resulting in discrete biological functions.^38^ A prevailing challenge with existing HDAC inhibitors lies in their limited specificity, as they often target multiple HDACs. Designing an isoform-selective inhibitor is challenging owing to the structural and chemical similarity of HDAC active sites. Owing to their lack of specificity, HDAC inhibitors may induce broad disruptions in normal cellular homeostasis, potentially accounting for the notable adverse effects observed in patients receiving HDAC inhibitor treatment.^13,53^ This underscores the proposition that rather than directly targeting HDAC enzymes with inhibitors, a more efficacious approach may entail modifying specific complexes. PFKL functions as a pivotal component within the Class I HDAC complex. Targeting either HDAC complexes or HDAC-associated proteins such as PFKL could selectively impact HDAC enzymatic activities, finely regulating epigenetic modifications, and thereby enhancing antitumor efficacy and broadening the clinical indications. The development of the PFKL-552-572-R8 peptide in our work represents a promising approach, warranting further investigation in future clinical trials.

## Materials and methods

### Ethics approval statement

All animal procedures were conducted in strict accordance with a protocol approved by the Ethical Committee of the National Center for Liver Cancer (NCLC, EDWLL-010), Shanghai.

This study involved human participants and was approved by the Ethical Committee of Eastern Hepatobiliary Surgery Hospital (No. EHBHDW-2022114). Participants gave informed consent to participate in the study before taking part.

### Cell lines

Human lung cancer cell lines (A549 and NCI-H596), the human CCA cell line RBE, human breast cancer cell lines (MCF-7 and BT-549), human ovarian cancer cell lines (RMG-I and TOV21G), and HEK-293T cells were purchased from the Shanghai Cell Bank of the Chinese Academy of Sciences, China. The human primary cell lines 1405R3, 783C-6, and 4786 R were isolated by our group. The human CCA cell line SK-CHA-1 was provided by H. You, Xiamen University. The TFK1 and HuCCT1 cell lines were provided by S.-Q. Zou, Tongji Hospital, Huazhong University of Science and Technology. A549 cells were cultured in F-12K medium supplemented with 1% glutamine. MCF-7 cells were cultured in MEM supplemented with 1% glutamine, 1% sodium pyruvate, and 0.01% recombinant human insulin. TOV21G was cultured in a mixed medium of MCDB105 (containing 1.5 g/L sodium bicarbonate) and Medium199 (containing 2.2 g/L sodium bicarbonate) at a 1:1 ratio. All human CCA cell lines, BT-549, and NCI-H596, were cultured in RPMI 1640 (Gibco). HEK293T and RMG-I cells were cultured in Dulbecco’s modified Eagle’s medium (DMEM; Gibco). All culture media were supplemented with penicillin (100 IU/mL; Gibco), streptomycin (100 μg/mL; Gibco), and 10% fetal bovine serum (Gibco). The cells were maintained at 37 °C and 5% CO_2_.

### Isolation and cultivation of primary tumor cells

Tumor tissues were collected and mechanically dissociated under aseptic conditions, followed by incubation with collagenase I (10 mg/mL; Sigma-Aldrich) and trypsin (0.10%; Sigma-Aldrich) at 37 °C for 1 h. Samples were filtered through a sterile 100-mm strainer. Red blood cells were eliminated using red blood cell lysis buffer (eBioscience). Freshly isolated primary tumor cells were maintained on Matrigel-coated culture plates and cultured in RPMI 1640 medium supplemented with 20% fetal bovine serum (Gibco), 1 μM dexamethasone (Sigma-Aldrich), 2 mM L-glutamine (Invitrogen), epidermal growth factor (EGF) (40 ng/mL) (PeproTech), fibroblast growth factor (FGF) (20 ng/mL) (PeproTech), insulin (5 μg/mL) (Sigma-Aldrich), penicillin (100 IU/mL) (Gibco), and streptomycin (100 μg/mL) (Gibco).

### Mice

BALB/c nu/nu mice (6 weeks old, males) were purchased from GemPharmatech Co., Ltd. NSG (NOD/SCID *Rag*2^-/-^, *IL2rg*^-/-^) mice (6 weeks old, males) were purchased from Shanghai Model Organisms Center, Inc. All the mice were housed in a specific pathogen-free (SPF) environment.

### Animal experiments

For cell-derived xenograft (CDX) models, tumor cells were resuspended in saline and combined with an equal volume of Matrigel. A total of 1 × 10^6^ cells in a 0.1 ml mixture were injected subcutaneously into the right flank of 6-week-old male nude mice. Once the tumor volume reached approximately 100 mm³, the mice were randomly grouped and treated with drugs or saline. The tumor volume was measured every 3 days and calculated via the following formula: tumor volume = (length × width^2^) × 0.5. When the tumor volume reached 1500 mm³, the mice were euthanized via CO_2_ asphyxiation. The tumor tissues were harvested for further study.

For patient-derived xenograft (PDX) models, fresh tumor samples were cut into small pieces of approximately 1 mm³ and subcutaneously transplanted into the flanks of NSG mice (P1 generation). After 2 to 3 months of tumor growth, the established PDX tumors were transplanted into subsequent generations (P_2_-P_n_) under the skin of nude or NSG mice. When the tumors reached approximately 100 mm^3^, the mice were randomly allocated to different groups and treated with HDAC inhibitors or saline. Tumor volume calculation, euthanasia of the mice, and specimen preparation followed the procedures of the CDX model.

### Compounds and antibodies

DMSO (HY-Y0320), romidepsin (HY-15149), panobinostat (HY-10224), vorinostat (HY-10221), and belinostat (HY-10225) were purchased from MedChemExpress. Ferrostatin-1 (S7243), gemcitabine (S1714), oxaliplatin (S1224), paclitaxel (S1150), Z-VAD-FMK (S7023), N-acetylcysteine (S1623), necrostatin-1 (S8037), lonidamine (S2610), 2-DG (S4701), 3-BP (S5426), penfluridol (S4151), LDC7559 (S9622) and shikonin (S8279) were purchased from Selleck Chemicals.

ROS Assay Kit (S0033) and the GSH and GSSG Assay Kit (S0053) were purchased from Beyotime. Lactate Assay Kit-WST (L256) was purchased from DOJINDO Laboratories. Annexin V-FITC Apoptosis Detection Kit (640914) was purchased from Biolegend. The Seahorse XF Glycolytic Rate Assay Kit (103344-100) was purchased from Agilent. Phosphofructokinase (PFK) activity assay kit (D799442) was purchased from Sangon Biotech (Shanghai). Cell viability assay kit (DD1101) and CUT&Tag assay kit (TD903) were purchased from Vazyme Biotech. Histone Acetyltransferase (HAT) activity assay kit (P-4003-96) was purchased from EpigenTek. Histone Deacetylase (HDAC) activity assay kit (AAT-13601) was purchased from AAT Bioquest. Zinc Quantification Kit (ab176725) was purchased from Abcam. The GST Protein Interaction Pull-Down Kit (21516) was purchased from Thermo Fisher Scientific.

Antibodies against cleaved caspase-3 (9664), PARP (9532), p-H2A.x (9718), MTA1 (5647), LSD1 (4218), acetylated lysine (9441), HDAC1 (34589), HDAC2 (5113), and HDAC3 (3949) were purchased from Cell Signaling Technology. Antibodies against histone H3 (A2348), H3K9ac (A7255), H3K27ac (A7253), HDAC5 (A0632), HDAC7 (A7285), HDAC9 (A2226) and HDAC11 (A6140) were purchased from ABclonal. An antibody against L-lactyl lysine was purchased from PTM BIO (PTM-1401RM). Antibodies against IgG (sc-66931) and PFKL (sc-393713) were purchased from Santa Cruz Biotechnology. Antibodies against GAPDH (60004-1-Ig), β-actin (81115-1-RR), GST-tag (10000-0-AP), His-tag (66005-1-Ig), HDAC1 (66085-1-Ig), HDAC3 (10255-1-AP), HDAC8 (17548-1-AP), HDAC4 (17449-1-AP), HDAC6 (12834-1-AP), HDAC10 (24913-1-AP), DNTTIP1 (11637-1-AP), SDS3 (25845-1-AP), TBL1X (13540-1-AP), CoREST (27686-1-AP), RBBP4 (20364-1-AP), Flag-tag (66008-4-Ig) and Myc-tag (16286-1-AP) were purchased from Proteintech. Antibodies against PFKM (111597) and PFKP (107857) were purchased from GeneTex. Antibodies against GPS2 (ab153986), PFKL (ab181064), Ki67 (ab15580), and CK19 (ab133496) were purchased from Abcam. The antibody against phospho-HDAC1 (PA5-36911) was purchased from Thermo Fisher Scientific.

### High-throughput drug screening and drug selection

To identify novel therapeutics against CCA, we conducted a high-throughput screen employing PerkinElmer’s integrated robotic Explorer G3 workstations. Our compound library comprised 164 FDA-approved or emerging agents. Following dilution to five specified concentrations, drugs were dispensed into 96-well plates in the sequence detailed in Supplementary Fig. 1a. The CCA cell lines were subsequently plated in 384-well plates at a density of 3000 cells per well in a 20 μL total volume. After a 24-h incubation, the compounds were added to the wells and incubated for 72 h. We then quantified cell viability via the CellTiter-Glo luminescent assay according to the manufacturer’s protocol. Signal detection was carried out on a Synergy2 plate reader operated by Gen5 software (BioTek). For data processing, the raw luminescence data were processed through the Breeze pipeline for curve fitting, yielding quality control metrics (e.g., Z-factors) and drug response parameters (AUC and DSS), as adapted from previous work.^54^ Drug responses were evaluated on the basis of the drug sensitivity score (DSS), and a cutoff of ≥10 was used to define efficacy. The complete drug library and screening dataset are available in Supplementary Tables 1-3.

### Genome-wide CRISPR screen and data analysis

The experiments were performed as previously described.^55,56^ Briefly, a human genome-wide lentiviral sgRNA library (approximately 1,23,411 sgRNAs targeting 20914 human genes) was used for screening. The library contained 6 sgRNAs against each protein-coding gene and 4 sgRNAs against each miRNA, along with 1000 nontargeting controls. Through lentiviral transduction, the genome-wide CRISPR library was transformed into TFK1 and 783C-6 cells (three independent replicates). After puromycin selection (2 μg/mL), 5 × 10^7^ cells were treated with either DMSO (*T*_untreated_) or HDAC inhibitors (*T*_treated_) for 7 days. Genomic DNA was extracted via a gDNA isolation kit (Qiagen, Germany). The sgRNA sequences were amplified via PCR followed by next-generation sequencing by Novogene Co., Ltd. A differential test between *T*_treated_ and *T*_untreated_ for each sgRNA was conducted by DESeq2. The sgRNA prioritization was represented by the ranking values calculated using the MAGeCK Robust Rank Algorithm. The analysis data are provided in Supplementary Tables 8–11.

### Detection of intracellular romidepsin

The quantification of intracellular romidepsin in the cell lysates was performed via the LC-MS/MS-based method. Briefly, appropriate amounts of romidepsin standard were weighed and dissolved to prepare a single standard stock solution. The cell lysates were pretreated, and 200 μL of the supernatant was transferred to a detection vial. The chromatographic conditions used were as follows: ACQUITY UPLC^®^ BEH HILIC column (2.1 × 100 mm, 1.7 μm, Waters Corporation); the injection volume was 5 μL; the column temperature was 40 °C; mobile phase A consisted of 50% acetonitrile and water (containing 0.1% formic acid); and mobile phase B consisted of 90% acetonitrile and water (containing 0.1% formic acid). Gradient elution: 0–6.5 min, 10–30% A; 6.5–7 min, 30–100% A; 7–14 min, 100% A; 14–14.5 min, 100–10% A; 14.5–17.5 min, 10% A. Flow rate: 0.3 mL/min. The mass spectrometry conditions were as follows: electrospray ionization (ESI) source and positive ionization mode. The ion source temperature was 500 °C, the ion source voltage was 5500 V, the collision gas pressure was 6 psi, the curtain gas pressure was 30 psi, and both the nebulizer gas and auxiliary gas pressure were 50 psi. Multiple reaction monitoring (MRM) was used for scanning. Data analysis: LC-MS detection of each working standard solution was performed, and a standard curve was plotted with the concentration of the working standard solution as the horizontal coordinate and the peak area as the vertical coordinate. The limit of quantification was determined using the signal-to-noise ratio method. The concentration at a signal-to-noise ratio of 10:1 (S/N = 10) is generally used to determine the limit of quantification. The quantitative analysis of all the samples was conducted based on the established sample pretreatment methods and instrument analysis methods.

### GST pull-down assay

Recombinant His-HDAC1, His-HDAC2, His-HDAC3, His-HDAC8, and GST-PFKL proteins were purified from the *Escherichia coli* BL21 (DE3) strain. The GST pull-down assay was conducted using the GST Protein Interaction Pull-Down Kit (Thermo) with the following steps: Equilibration of glutathione agarose beads: Pull-Down buffer was mixed with TBS at a 1:1 ratio to prepare washing buffer. The appropriate amount of agarose beads was placed in a tube and washed five times. The GST-tagged protein was incubated with magnetic beads: an appropriate amount of GST-PFKL protein was added to the washed magnetic beads, which were subsequently incubated at 4 °C with rotation for 1 h, followed by five washes via centrifugation. Capture of His-tagged protein: An appropriate amount of His-HDAC protein was added to the magnetic beads and incubated at 4 °C with rotation for 1.5 h, followed by five washes via centrifugation. Protein elution: Glutathione elution buffer was prepared, added to the magnetic beads, and incubated with rotation for 5 min. The eluted protein was collected in a 1.5 ml tube. Next, 2×SDS loading buffer was added to the beads, which were subsequently boiled for 5 min. The samples were analyzed by SDS-PAGE and immunoblotting.

### SPR analysis

The interactions between His-HDAC1, His-HDAC2, His-HDAC3, His-HDAC8, and GST-PFKL were analyzed using the OpenSPRTM system (Nicoya) at 25 °C. Recombinant His-HDAC proteins were immobilized on a sensor chip (NTA, Nicoya) using an amine coupling kit (Nicoya). The final His-HDACs immobilized abundance was typically ~16000 RU. Subsequently, GST and GST-PFKL were injected as analytes at different concentrations. The PBS-P buffer used for the experiments contained 10 mM phosphate buffer, 137 mM NaCl, 2.7 mM KCL, and 0.05% surfactant P20. To detect the binding affinity between His-HDACs and GST-PFKL, analytes at the corresponding concentrations were injected at a flow rate of 20 μL/min, with a contact time of 240 s and a dissociation time of 360 s. Finally, the chip platforms were washed with buffer and 50% dimethyl sulfoxide. TraceDrawer software was used to analyze the data through curve fitting with a 1:1 binding model.

### HAT and HDAC enzyme activity assays

The EpiQuik HAT activity/inhibition assay kit was used to detect the HAT enzyme activity. The assay was performed as follows: (i) Preparation of nuclear extracts from the samples for subsequent detection of HAT activity. The HAT standard was diluted to different concentrations using wash buffer. Fifty microliters of diluted HAT substrate was mixed and added to each well, followed by incubation at room temperature for 30–45 min. (ii) Removal of liquid from the wells, followed by washing three times with wash buffer. Then, 26 μL of detection buffer, 2 μL of diluted acetyl-CoA, and 2 μL of sample nuclear extract (4–20 μg) were added to each well, mixed and incubated at 37 °C for 30–60 min. Liquid in the plate was discarded, followed by three washes. 50 μL of diluted capture antibody was added to each well, and incubated at room temperature with shaking for 60 min. (iii) Three repeated washes were performed, followed by adding 50 μL of diluted detection antibody to each well, and incubating at room temperature for 25–30 min. Three repeated washes were performed, and 100 μL of developing solution was added to each well. The plate was then incubated in the dark at room temperature for 2–10 min. Fifty microliters of stop solution was added to each well to terminate the reaction. The color should turn yellow, and absorbance was read at 450 nm wavelength to draw the standard curve and calculate the corresponding HAT enzyme activity.

A HDAC enzyme activity assay kit (AAT Bioquest) was used to detect HDAC activity in cells, as well as the enzymatic activity of the purified HDAC protein. The detection steps were as follows: (i) Collection of cell lysates or purified proteins for subsequent HDAC activity detection. Forty microliters of the sample to be tested and 10 μL of detection buffer were added to each well, mixed and incubated at room temperature or 37 °C for 10–20 min. (ii) 50 µL of substrate working solution was added directly to each well, followed by incubation at room temperature or 37 °C for 30–60 min. (iii) Fluorescence intensity was measured at Ex/Em = 490/525 nm, and HDAC enzyme activity was calculated.

### Immunoblot analysis

For immunoblotting, cells were washed with ice-cold PBS and lysed in RIPA lysis buffer (supplemented with protease inhibitor and phosphatase inhibitor cocktails Ⅱ and Ⅲ) for 15 min at 4 °C. The protein concentrations of the samples were normalized using a BCA protein assay kit (Thermo). Immunoblots were performed with specific primary antibodies, followed by the addition of a fluorescein-conjugated secondary antibody, after which the proteins were detected via an Odyssey fluorescence scanner (LI-COR).

### Cell proliferation and viability assay

The cell proliferation rates were determined with a CCK-8 Cell Counting Kit (Vazyme, A311) in strict accordance with the provided protocol. The colony formation capacity was assessed by plating the cells in 6-well plates (5000 cells/well), followed by drug treatment over a 7-day period. Finally, the resulting colonies were subjected to crystal violet staining for subsequent quantification.

Cell viability was assessed using the CellTiter-Glo luminescent cell viability assay (Vazyme, DD1101) following the manufacturer’s instructions. Briefly, 5000 cells per well were seeded into a 96-well white bottom permeable plate. Different concentrations of drugs were added to each well. After 72 h of treatment, a mixture of CellTier reagent and serum-free culture medium was prepared at a 1:1 ratio, and 100 μL of the mixture was added to each well. After incubation at 37 °C for 15 min, the luminescence was measured using a microplate reader (Synergy H1, Biotek). All the experiments were conducted in triplicate. Cell viability (relative luminescence) was defined as the luminescence of the drug treatment relative to that of the vehicle control. The GI50 (concentration causing 50% growth inhibition) was calculated with GraphPad 9.0 via a nonlinear regression model.

### Immunohistochemistry (IHC) and Immunofluorescence (IF)

Formalin-fixed, paraffin-embedded (FFPE) tissue sections (4-μm thick) were prepared for histological examination by hematoxylin and eosin (H&E) staining or for immunohistochemical (IHC) analysis. For IHC, after deparaffinization and rehydration, endogenous peroxidase activity was inhibited by treatment with 3% H₂O₂, and nonspecific binding sites were blocked with 1% bovine serum albumin (BSA). The sections were then probed with a primary antibody at 4 °C overnight. Subsequently, an HRP-labeled secondary antibody was applied, and the samples were incubated for 1 h at 37 °C. Antigen-antibody complexes were visualized using a 3,3′-diaminobenzidine (DAB) substrate. Finally, the nuclei were counterstained with hematoxylin, and the sections were dehydrated, cleared, and mounted with a permanent mounting medium. For IF, cells were incubated with 4% formaldehyde, 0.5% Triton X-100, 5% BSA in PBS, primary antibodies, Alexa Fluor 546- or 488-conjugated secondary antibodies (Invitrogen), and DAPI. Images were acquired via a Leica SP8 confocal microscope (Leica).

### RNA extraction and qPCR analysis

Total RNA was isolated using TRIzol Reagent (Invitrogen), 2 μg of mRNA was used to synthesize cDNA using the HiScript III RT SuperMix (Vazyme, R323-01). qPCR was performed using SYBR Green Supermix (Roche), and the results were normalized to 18S or GAPDH (glyceraldehyde-3-phosphate dehydrogenase) control. The primers used are listed in Supplementary Table 12.

### Plasmid, lentivirus, and siRNA transfection

The Flag-HDAC1-WT, Flag-PFKL-WT, and MYC-PFKL-WT plasmids were purchased from SinoBiological. The plasmids for HDAC1 (D176A, H178A, D176A + H178A, ∆D176, ∆H178, ∆D176 + H178, and ∆D181) were constructed on the basis of the corresponding wild-type plasmids. Transient transfection of the plasmids into the cell lines was performed via jetPEI DNA transfection reagent (Polyplus).

The lentiviruses used for PFKL interference (shPFKL-1, shPFKL-2), PFKL overexpression (ovPFKL), and sgRNA (NC, SgPFKL-1, SgPFKL-2) were constructed by GeneChem, Shanghai. shRNA- and sgRNA-expressing vectors were introduced into the cancer cell lines through lentiviral infection. For lentivirus infection, appropriate viral solutions were added to medium containing polybrene (4 μg/mL). After 48 h of infection, the cells were selected with puromycin (2 μg/mL) and PFKL expression was tested via qPCR or immunoblotting.

For siRNA transfection, Hieff Trans siRNA/miRNA Transfection Reagent (Yeasen Biotechnology) was used following the manufacturers’ protocols. siRNA oligos were designed and synthesized by GenePharma, Shanghai. The siRNA, shPFKL, and sgPFKL sequences are listed in Supplementary Table 13.

### Immunoprecipitation (IP)

The cells were collected in lysis buffer 150 mM NaCl, 25 mM Tris-HCl, 0.5% NP-40, and protease and phosphatase inhibitors were added before use. The lysate was centrifuged and immunoprecipitated with protein A/G magnetic beads (Enriching Biotechnology) and primary antibodies. For immunoprecipitation of FLAG-tagged proteins, anti-FLAG-M2 magnetic beads (M8823, Sigma Aldrich) were used according to the manufacturer’s instructions. The initial whole-cell lysates and precipitated proteins were boiled in SDS-PAGE buffer, separated by SDS-PAGE, and detected by immunoblotting.

### Eukaryotic protein purification via the FLAG tag

Flag-tagged proteins were purified from HEK-293T cells via anti-FLAG M2 magnetic beads (M8823, Sigma). The cells were lysed in RIPA buffer and incubated overnight at 4 °C with anti-FLAG M2 magnetic beads for immunoprecipitation. The samples were washed three times with lysis buffer, and the FLAG-tagged proteins were eluted with 3×FLAG peptide (180 μg/mL, F4799, Sigma) at 4 °C for 2–3 h. Subsequently, the FLAG-tagged proteins were dialyzed for 4 h at 4 °C in dialysis buffer (20 mM HEPES, 50 mM NaCl, 0.4% glycerol, pH 7.3) via a 10 kDa dialysis bag. After dialysis, the purified proteins were transferred to EP tubes and stored at −80 °C for immunoblotting and enzymatic activity assays.

### CUT&Tag library construction

The CUT&Tag assay was conducted in accordance with the manufacturer’s protocol via the Hyperactive™ In-Situ ChIP Library Prep Kit for Illumina (TD901, Vazyme Biotech). In brief, concanavalin A-coated magnetic beads (ConA beads) were incubated with resuspended cells to facilitate cell attachment. Cells were then permeabilized with digitonin to enable antibody access. Subsequent incubations were performed sequentially: first with a combination of rabbit anti-H3K9ac (A7255, ABclonal) and anti-H3K27ac (A72535, ABclonal) primary antibodies, followed by a species-appropriate secondary antibody, and finally with the Hyperactive pA-Tn5 transposase complex. This approach ensures that the transposase is specifically targeted to genomic regions associated with the histone modifications of interest. Tagmentation was initiated by the pA-Tn5 transposase, which simultaneously cleaves DNA and ligates pre-annealed Illumina P5 and P7 adapters at these targeted sites. The adapter-ligated DNA fragments were then amplified by PCR to generate the sequencing libraries. Library quality control was performed via an Agilent 2100 Bioanalyzer to assess size distribution and concentration. Libraries passing QC were sequenced on an Illumina NovaSeq 6000 platform by Lc-Bio Technology Co., Ltd (Hangzhou, China), yielding 150-bp paired-end reads for subsequent bioinformatic analysis.

### Seahorse assay

The real-time extracellular acidification rate (ECAR) was measured using the Seahorse XF Glycolytic Rate Assay Kit (Agilent, 103344-100) on the Seahorse XF 96 Extracellular Flux Analyzer (Seahorse Bioscience, North Billerica, MA, USA) according to the manufacturer’s instructions. Briefly, 1×10^4^ wild-type or PFKL-deficient TFK1 or 783C-6 cells were seeded in an XF96 cell culture microplate. The final concentrations of rotenone/antimycin A (Rot/AA) and 2-deoxy-D-glucose (2-DG) were 0.5 μM and 50 mM, respectively.

### Structure preparation and molecular docking

The three-dimensional (3D) structure of the compound reduced-romidepsin was constructed with energy minimization in the MMFF94 force field. The crystal structures of the proteins HDAC1 and PFKL (PDB codes: 4BKX and 7LW1) were obtained from the RCSB Protein Data Bank (http://www.rcsb.org/). The 3D structure of the peptide (CDRIKQSASGTKRRVFIVETM) was derived from the PFKL protein with energy minimization in the AMBER ff19SB^57^ force field.

### The ternary complex of HDAC1, reduced-romidepsin, and PFKL

Initially, the binding pocket of HDAC1 was defined by residues Asp176 and His178. The DOCK 6.9 program^58^ was employed to perform semiflexible docking between HDAC1 and reduced-romidepsin. The energy evaluation was carried out using the Grid scoring function, and the HDAC1-reduced-romidepsin conformation with the best score was obtained. The binding pocket of this conformation was then defined by residue numbers 170-183. Protein-protein docking of HDAC1-reduced-romidepsin and PFKL was conducted in HDOCK SERVER.^59^ Finally, ten expected ternary complexes were obtained for molecular dynamics simulations.

### The ternary complex of HDAC1, reduced-romidepsin, and peptide

On the basis of the HDAC1-reduced-romidepsin conformation, the binding pocket was defined in the same way. Subsequently, protein-peptide docking was performed between HDAC1-reduced-romidepsin and the peptide in ZDOCK SERVER.^60^ Finally, one expected ternary complex was obtained for molecular dynamics simulations.

### Molecular dynamics simulations

#### The ternary complex of HDAC1, reduced-romidepsin, and PFKL

The receptor was prepared, and missing atoms of residues were fixed by the advanced PDB-Preparation tool in the Yinfo Cloud Computing Platform (https://cloud.yinfotek.com/) via PDBFixer and the tLEaP module in AmberTools 20. The AM1-BCC charges were calculated for the reduced-romidepsin compound via the Amber antechamber program.^61^ The MCPB method was subsequently used to construct the topological parameters of the metal complex.^62^

The simulation system was solvated in a cubic water box employing the OPC water model, extending 10 Å beyond the solute in all dimensions. Periodic boundary conditions (PBC) were applied, and the system’s net charge was neutralized with Na⁺ ions. For the calculation of nonbonded interactions, van der Waals forces were described by a Lennard‒Jones 12‒6 potential with a cutoff distance of 10 Å, and long-range electrostatic interactions were handled with the particle mesh Ewald (PME) method. All covalent bonds involving hydrogen atoms were constrained via the SHAKE algorithm. To eliminate steric clashes, the system underwent energy minimization in two stages: first, with 2500 steps of steepest descent plus 2500 steps of conjugate gradient minimization, applying positional restraints of 10.0 kcal/(mol Å²) on all nonhydrogen atoms; second, full minimization without restraints for 10,000 steps each of steepest descent and conjugate gradient. Following minimization, the system was gradually heated from 0 to 300 K over 20 ps in the NVT ensemble. This was followed by a two-step equilibration protocol: (1) a 200-ps NPT simulation with restraints on heavy atoms and (2) a 1 ns NVT simulation with all restraints released. During equilibration, the temperature was maintained at 300 K via a Berendsen thermostat, and the pressure was regulated at 1 atm via a Monte Carlo barostat. A production MD simulation was then performed in the NVT ensemble for 2 ns using a 2 fs integration time step. The binding free energy was estimated for this trajectory via the Molecular Mechanics/Generalized Born Surface Area (MM/GBSA) approach.^63^ The system conformation demonstrating the most favorable binding free energy was selected for a final, extended 100 ns NVT production simulation. Following this simulation period, we identified the concluding frame of the ternary complex and removed the PFKL molecule, resulting in the formation of a binary complex. Then, another 100 ns NVT simulation was conducted following the aforementioned procedure.

To explore the influence of the HDAC1 protein on the noncovalent binding of the compound, conventional nonbonded molecular dynamics simulations with a duration of 20 ns were conducted for binary and ternary complexes. The binding free energies were calculated on the basis of the 20 ns MD trajectory. The root-mean-square deviation (RMSD) and distance were analyzed via the CPPTRAJ^64^ module, and clustering was performed via the DBSCAN algorithm.

#### The ternary complex of HDAC1, reduced-romidepsin, and peptide

A 50 ns NVT simulation of the ternary complex (referred to as complex A) was conducted according to the above method. Next, we identified the concluding frame of complex A and removed one residue of the peptide (Thr562 with a yellow background), resulting in the formation of complex B. Next, another 50 ns NVT simulation was conducted following the aforementioned procedure. Conventional nonbonded molecular dynamics simulations with a duration of 20 ns were subsequently conducted for complexes A and B. The binding free energies were calculated via the Molecular Mechanics Generalized Born (MM/PBSA) method on the basis of the last 6 ns of the MD trajectory. The root-mean-square deviation (RMSD), root-mean-square fluctuation (RMSF), hydrogen bonding, and distance were analyzed via the CPPTRAJ module, and clustering was performed via the DBSCAN algorithm. The salt bridges were analyzed via VMD.

#### Zinc quantification assay

Zinc in cells or purified proteins was quantified via a zinc quantification kit (Abcam, ab176725) according to the manufacturer’s instructions. The steps were as follows: standard solutions of 30 µM, 10 µM, 3 µM, 1 µM, 0.3 µM, 0.1 µM, and 0 µM were obtained by continuous dilution. The cells were lysed in EDTA-free lysis buffer and centrifuged to remove cellular debris. A total of 7% TCA solution was added to the cell lysate, and the mixture was rotated for 5 min to precipitate the proteins. The sample was subsequently neutralized with 1 M Na_2_CO_3_ and kept on ice. Zinc detection buffer was prepared; 50 µl of the sample and 50 µl of zinc detection buffer were added to each well, and the mixture was incubated at room temperature in the dark for 5–10 min. Zinc bound to a probe with enhanced fluorescence at Ex/Em 485/525 nm. This probe presented a high increase in fluorescence in response to zinc.

#### Quantification and statistical analysis

Statistical analysis was performed via GraphPad Prism 9. The data are presented as the means ± s.d.s of all the cell culture experiments and the means ± s.e.m.s of all the animal experiments. For all the experiments, more than three biological replicates were performed under the same conditions. An unpaired Student’s *t*-test (two-tailed) was used to define statistical significance when two groups were compared. One-way ANOVA was used to compare multiple mean values, and two-way ANOVA was used to compare multiple mean values across conditions. *P* < 0.05 was considered statistically significant, and the *P* values are indicated in the figures.

## Supplementary information

SUPPLEMENTAL MATERIAL

Supplementary Table 1-13

## Data Availability

We declare that data supporting the findings of this study are available within this manuscript and its supplementary information files. The sequencing data analyzed in this publication have been deposited in the China National Genomics Data Center (NGDC) and are accessible through accession numbers PRJCA046540 (RNA-seq data), PRJCA045203 (CUT&Tag data), PRJCA045193 (mass spectrometry data of HDAC1 and PFKL immunoprecipitates), and PRJCA046546 (ATAC-seq data).
