# Supplementary material for: The noncanonical function of liver-type phosphofructokinase potentiates the efficacy of HDAC inhibitors in cancer
Source: Signal Transduct Target Ther. 2025 Oct 14;10:341. doi: 10.1038/s41392-025-02443-0 (PMC12518634; doi:10.1038/s41392-025-02443-0)
Supplement: Supplementary file 1 — SUPPLEMENTAL MATERIAL [file 41392_2025_2443_MOESM1_ESM.docx]

Supplementary Materials for

The non-canonical function of glycolytic enzyme PFKL potentiates the efficacy of HDAC inhibitors in cancer

Taiyu Shang, Tianyi Jiang, Jiangqi Tan, Haolin Jiang, Mengyou Xu, Yufei Pan, Yunkai Lin, Xiaowen Cui, Chenxi Tian, Huibo Feng, Yibin Chen, Mengmiao Pei, Xin Geng, Shuqun Cheng, Yexiong Tan, Hongyang Wang, Liwei Dong

Correspondence to: dlw@smmu.edu.cn

**This PDF file includes:**

Supplementary Figures 1 to 16

Supplementary Tables 1 to 13

**Supplementary Fig. 1**

**
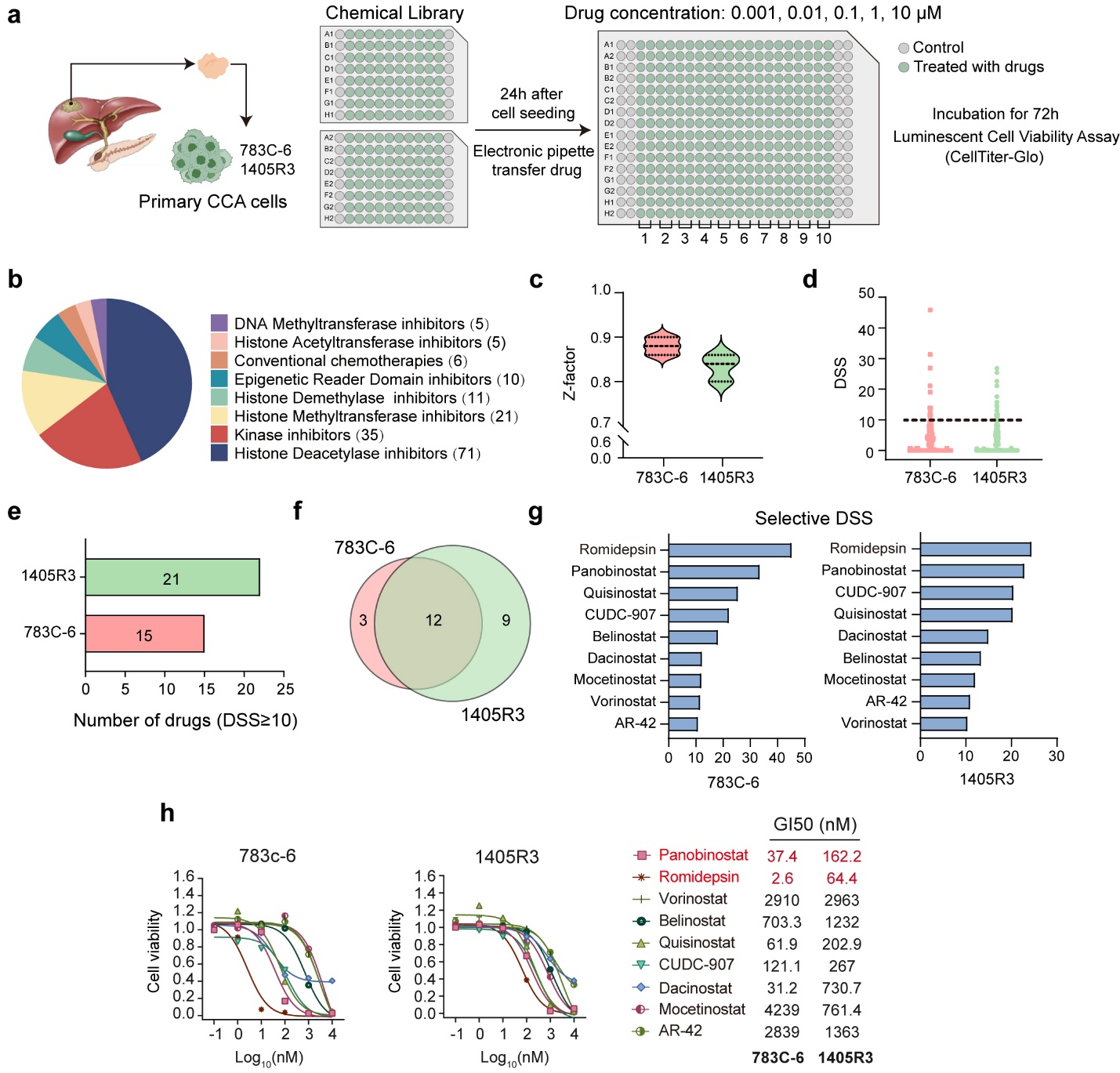
**

**Supplementary Fig. 1. A high-throughput drug screen to identify drugs targeting CCA. a** Schematic representation of the drug screening protocol. TFK1 and 783C-6 cells were seeded into 384-well plates and subjected to treatment with drugs for 72 hours. Each drug was tested at five different concentrations (10, 1, 0.1, 0.01, and 0.001 μM), and each treatment was replicated twice. **b** Classification of drugs used for screening. The quantity of each drug category is illustrated in the pie chart. **c** Z-factors representing the viability of each plate of CCA cell lines. **d** Drug sensitivity score (DSS) for each compound in the two CCA cell lines. **e** Counts of drugs associated with a DSS ≥ 10 in both CCA cell lines. **f** Venn diagram depicting the overlap of identified drugs with a DSS≥ 10 in the two CCA cell lines. A total of 12 drugs were identified with a DSS ≥ 10 in both cell lines. **g** Histogram illustrating the DSS of the 9 HDAC inhibitors among the 12 identified drugs. **h** Cell viability of 783C-6 and 1405R3 cells after treatment with various concentrations of various HDAC inhibitors for 72 hours.

**Supplementary Fig. 2**


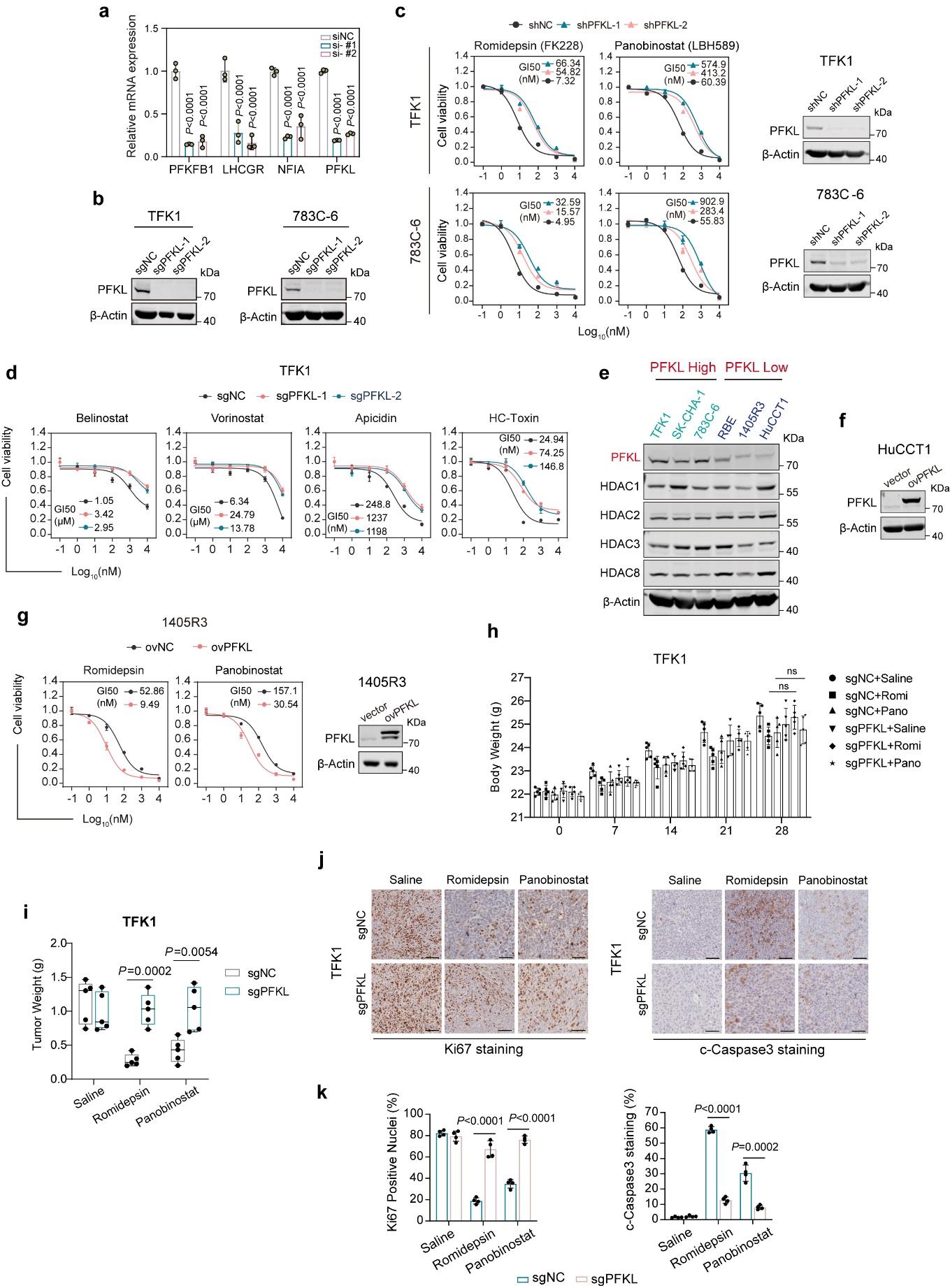


**Supplementary Fig. 2. PFKL enhances the efficacy of HDAC inhibitors in vitro and in vivo.**

**a** Quantitative PCR analysis of interference efficiency for the four candidate genes (*n* = 3 biological replicates, Student’s *t* test). **b** PFKL knockout efficiency was assessed via western blotting with sgRNAs. β-actin was used as a loading control. **c** Cell viability of TFK1 and 783C-6 cells with negative control (shNC) or PFKL knockdown (shPFKL) after treatment with specified concentrations of romidepsin or panobinostat for 72 hours. **d** Cell viability of TFK1 cells expressing either a negative control sgRNA (sgNC) or sgRNAs targeting PFKL (sgPFKL-1 and sgPFKL-2) following treatment with specified concentrations of belinostat, vorinostat, apicidin or HC-toxin for 72 hours. **e** Immunoblot analyses of the expression of PFKL, HDAC1, HDAC2, HDAC3 and HDAC8 in CCA cells. **f** PFKL overexpression efficiency was assessed by western blotting in HuCCT1 cells. β-actin was used as a loading control. **g** Cell viability of 1405R3 cells with vector or PFKL overexpression after 72 hours of treatment with the indicated concentrations of romidepsin or panobinostat. **h** Body weight of each group of TFK1-sgNC and TFK1-sgPFKL xenografts at the end of treatment is plotted (*n* = 5 mice per group, two-way ANOVA). **i** Tumor weights of each group of TFK1-sgNC and TFK1-sgPFKL xenografts at the end of treatment are plotted (*n* = 5 mice per group, Student’s *t* test). **j, k** Representative results and quantification of Ki67 and c-Caspase3 staining in TFK1-sgNC and TFK1-sgPFKL CDX tumors (*n* = 4 fields, Student’s *t* test). Scale bars, 100 μm. All the statistical data are presented as the means±SEMs.

**Supplementary Fig. 3**


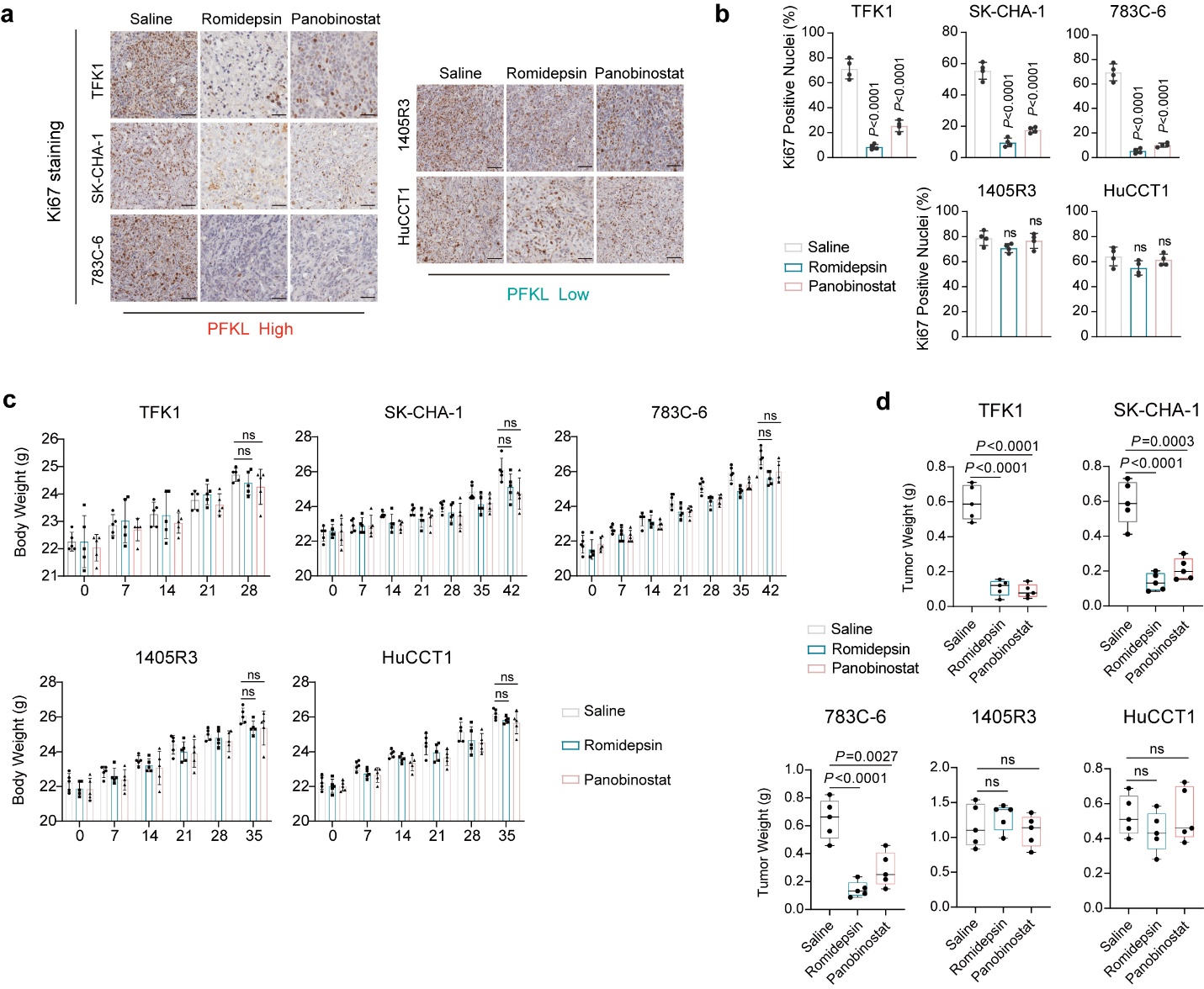


**Supplementary Fig. 3. PFKL facilitates the efficacy of HDAC inhibitors in CCA. a, b** Representative results and quantification of Ki67 staining in TFK1, SK-CHA-1, 783C-6, 1405R3, and HuCCT1 CDX tumors (n = 4 fields, Student’s *t* test). Scale bars, 100 μm. **c** Body weights of each group of TFK1, SK-CHA-1, 783C-6, 1405R3, and HuCCT1 xenografts at the end of treatment are plotted (*n* = 5 mice per group, two-way ANOVA). **d** Tumor weights of each group of TFK1, SK-CHA-1, 783C-6, 1405R3, and HuCCT1 xenografts at the end of treatment are plotted (*n* = 5 mice per group, Student’s *t* test). All the statistical data are presented as the means±SEMs.

**Supplementary Fig. 4**


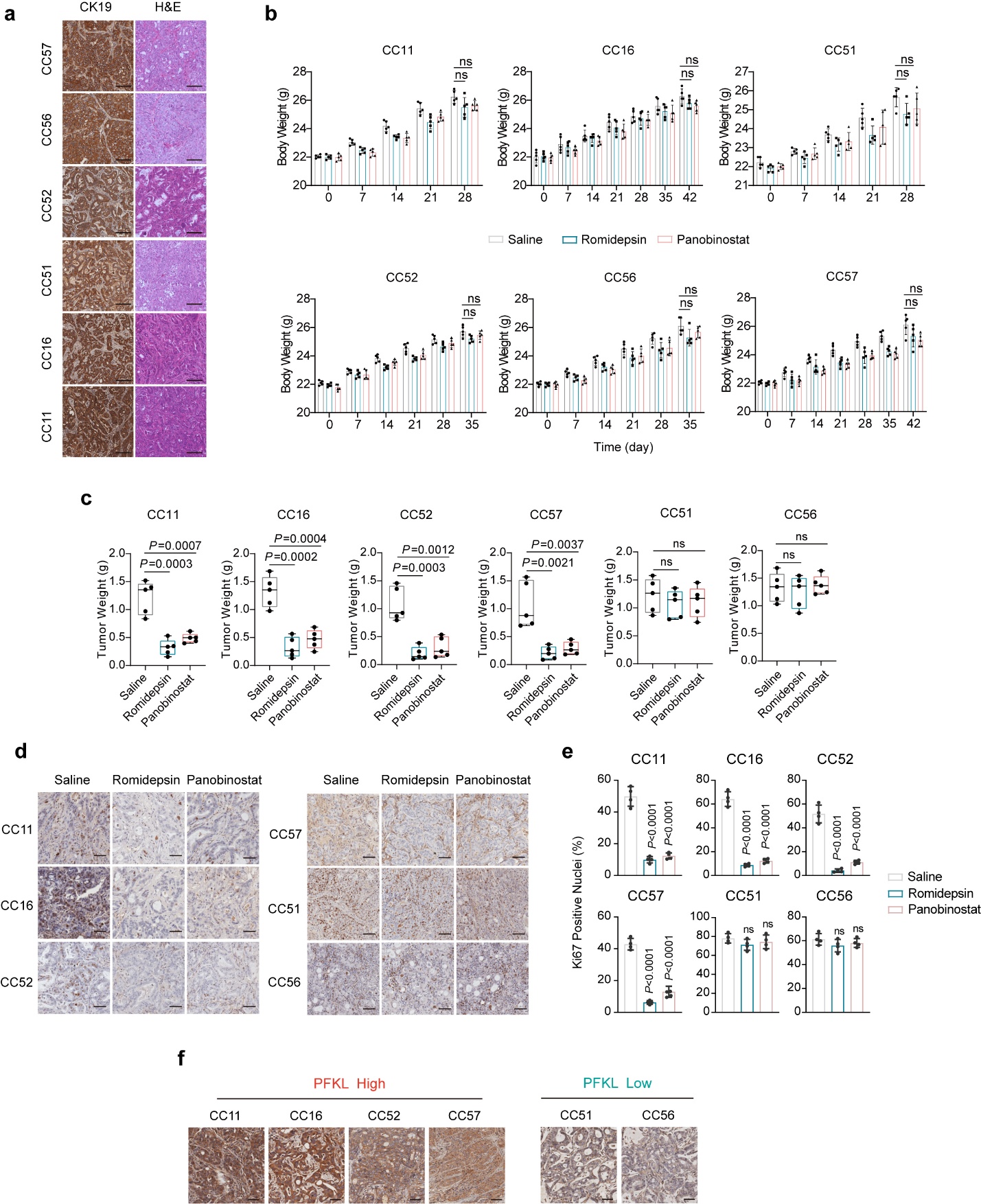


**Supplementary Fig. 4. PFKL serves as a predictor of the in vivo efficacy of HDAC inhibitors in CCA. a** Morphological and histopathological comparison of PDX models. Representative images of hematoxylin and eosin (H&E) and CK19 immunostaining are shown. Scale bars, 100 μm. **b** The body weights of each group of CC11, CC16, CC52, CC57, CC51 and CC56 tumors at the end of treatment are plotted (*n* = 5 mice per group, two-way ANOVA). **c** The tumor weights of each group of CC11, CC16, CC52, CC57, CC51 and CC56 tumors at the end of treatment are plotted (*n* = 5 mice per group, Student’s *t* test). **d, e** Representative images and quantification of Ki67 staining in PDX models (n = 4 fields, Student’s *t* test). Scale bars, 100 μm. **f** Representative images of PFKL staining in CCA PDX samples. The samples were categorized into “PFKL-High” or “PFKL-Low” groups on the basis of PFKL staining intensity. Scale bars, 100 μm. All the statistical data are presented as the means±SEMs.

**Supplementary Fig. 5**


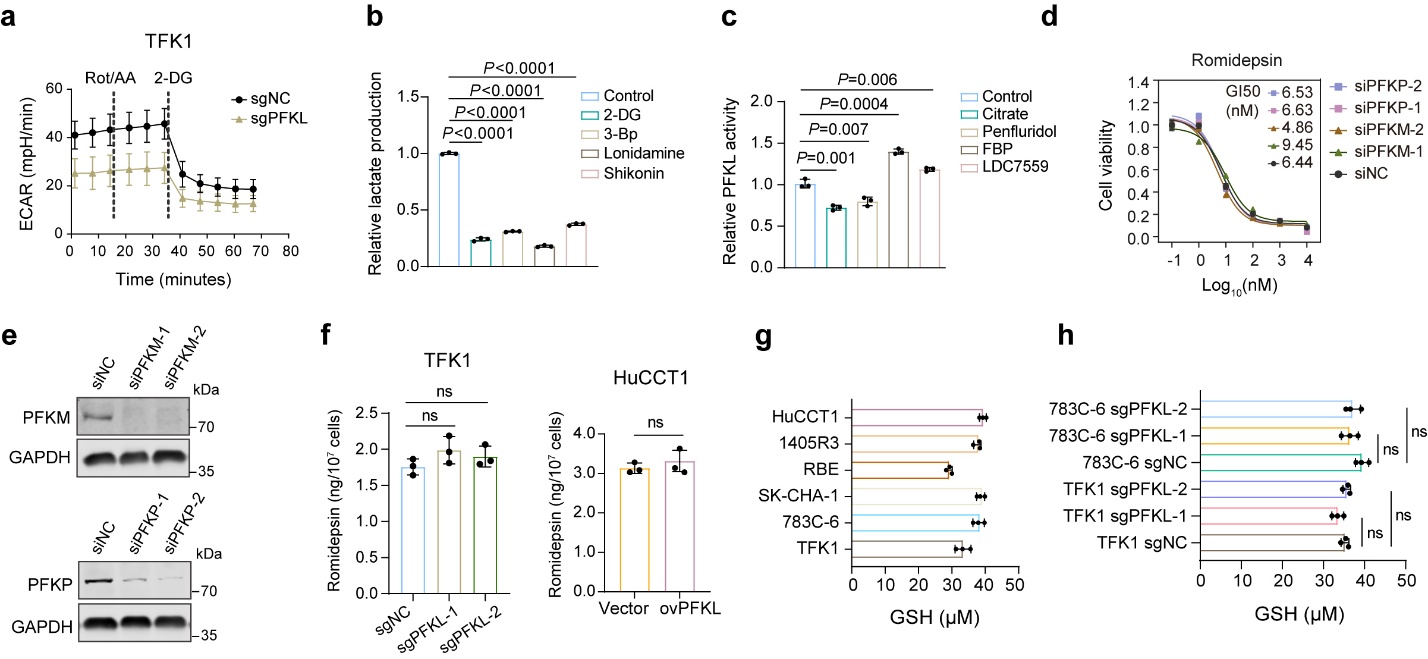


**Supplementary Fig. 5.** **PFKL enhances the efficacy of romidepsin independent of its metabolic function. a** Seahorse metabolic analysis (ECAR) of TFK1 cells expressing sgNC or sgPFKL (n=3 biological replicates). **b** Lactate production in TFK1 cells treated with 2-DG, 3-BP, lonidamine, or shikonin (n=3 biological replicates, Student’s *t* test). **c** PFKL enzymatic activity in TFK1 cells treated with citrate, penfluridol, FBP, or LDC7559 (n=3 biological replicates, Student’s *t* test). **d** The cell viability of TFK1 cells treated with specified concentrations of romidepsin for 72 hours after interference with PFKP or PFKM. **e** Immunoblot analyses of the interference efficiency of siRNAs targeting PFKM and PFKP. GAPDH was used as a loading control. **f** Intracellular abundance of romidepsin detected in PFKL-knockout (sgPFKL) TFK1 cells and PFKL-overexpressing (ovPFKL) HuCCT1 cells (n=3 biological replicates, Student’s *t* test). **g** Measurement of GSH levels in CCA cells (n=3 biological replicates). **h** Measurement of GSH levels in TFK1 and 783C-6 cells expressing negative control or sgRNA targeting PFKL (n=3 biological replicates, Student’s *t* test). All the statistical data are presented as the means±SEMs.

**Supplementary Fig. 6**

**
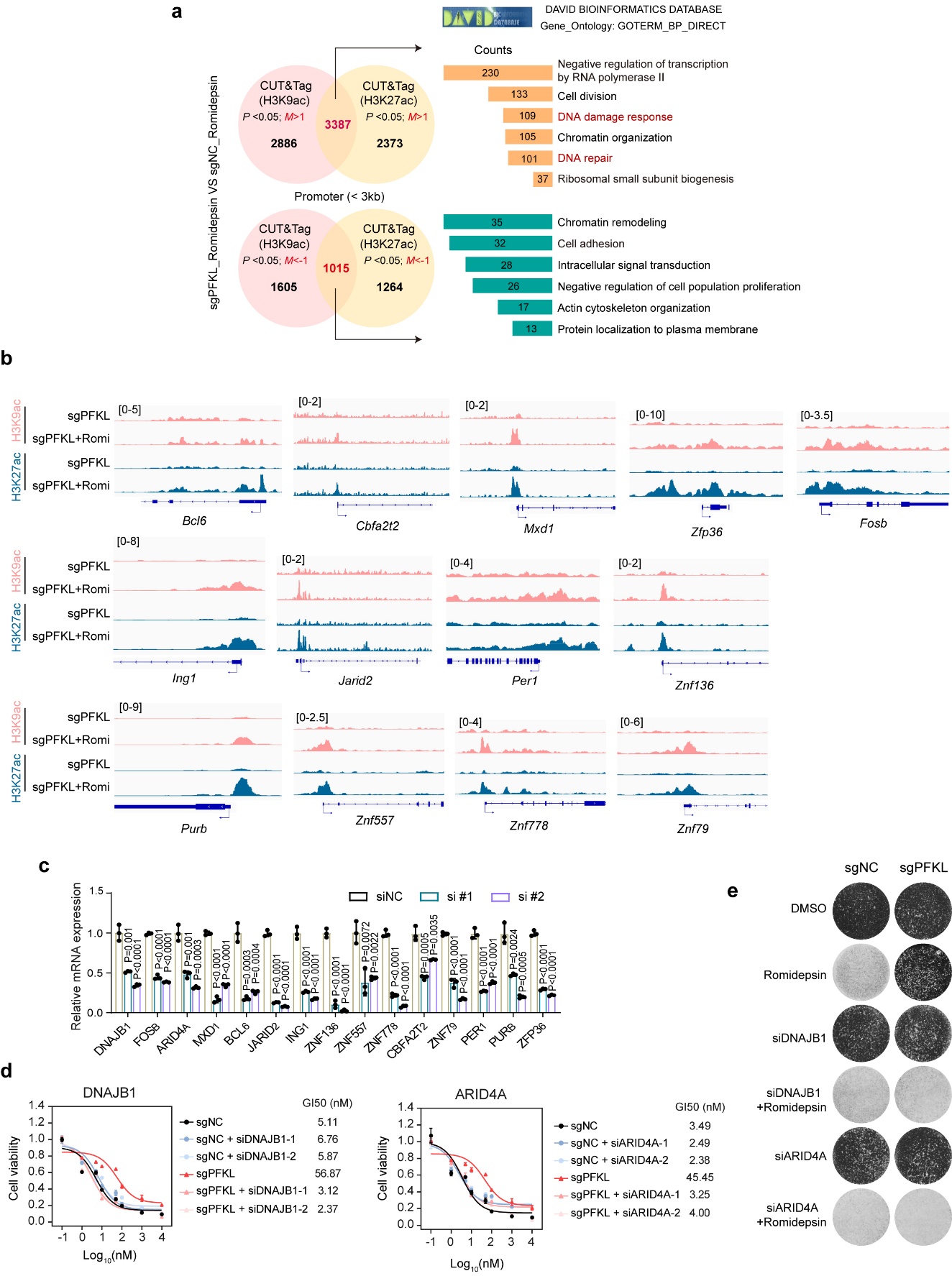
**

**Supplementary Fig. 6. PFKL deletion antagonizes the transcriptional activation induced by romidepsin. a** CUT&Tag detection of H3K9ac and H3K27ac and bioinformatics analysis via the DAVID database. **b** Normalized read densities for H3K9ac and H3K27ac at the candidate genes. **c** The interference efficiency of the candidate genes was detected via qPCR (n=3 biological replicates, Student’s *t* test). **d** Cell viability of TFK1-sgNC and TFK1-sgPFKL cells following treatment with specified concentrations of romidepsin for 72 hours after Dnajb1 and Arid4a interference. **e** Effects of Dnajb1 and Arid4a interference in TFK1-sgNC or TFK1-sgPFKL cells in a colony formation assay. The cells were treated with specified concentrations of romidepsin for 10 days. All the statistical data are presented as the means±SEMs.

**Supplementary Fig. 7**


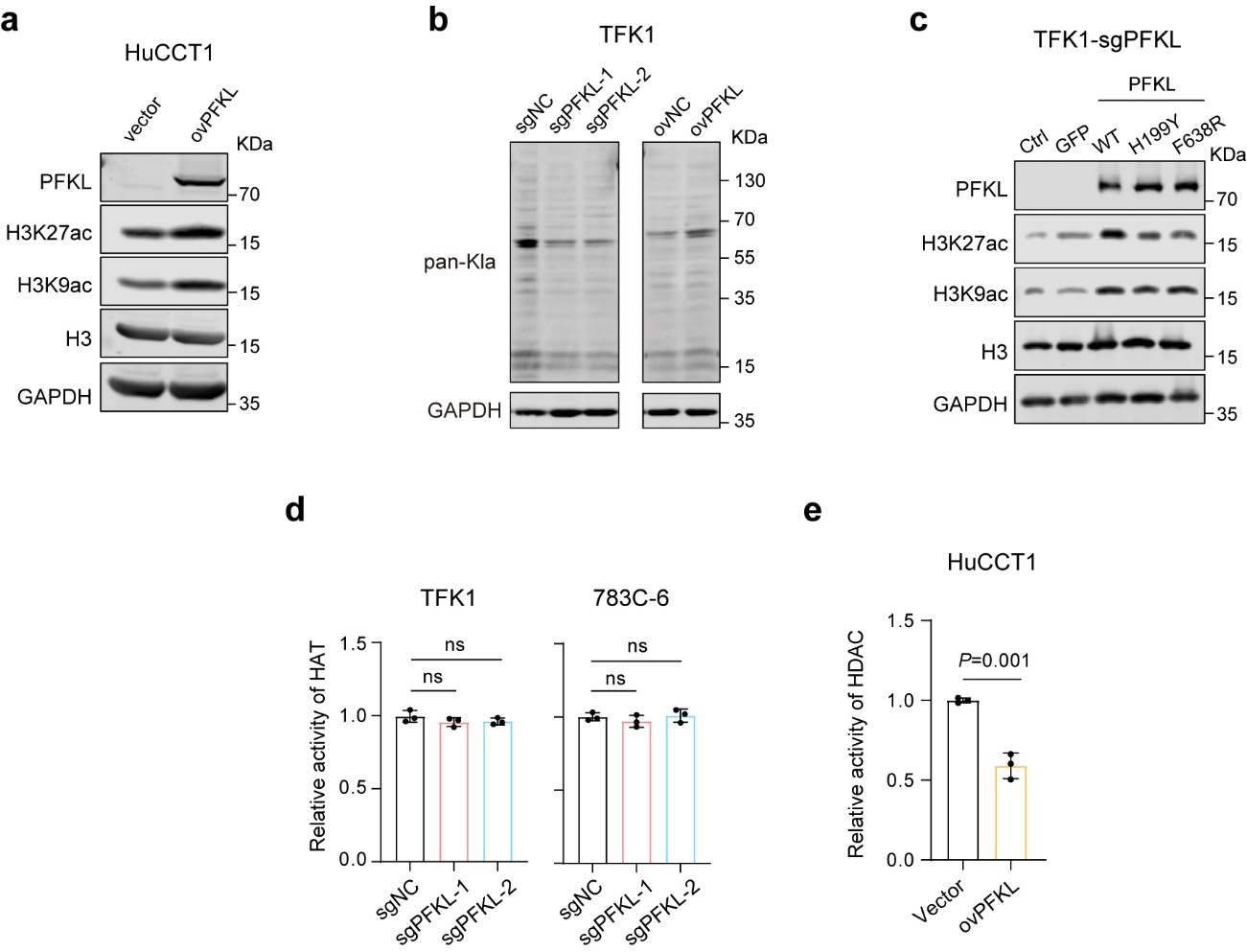


**Supplementary Fig. 7. PFKL suppresses HDAC enzymatic activity. a** Immunoblot analyses of the expression of H3K9ac and H3K27ac in HuCCT1 cells with vector or PFKL overexpression. **b** Immunoblot analysis of pan-Kla expression in TFK1 cells with differential expression of PFKL. **c** Immunoblot analyses of the expression of PFKL, H3K9ac and H3K27ac in PFKL-depleted TFK1 cells transfected with the GFP, PFKL-WT, PFKL-H199Y and PFKL-F638R plasmids. **d** Detection of HAT enzymatic activity in TFK1 and 783C-6 cells expressing sgNC or sgPFKL (n=3 biological replicates, Student’s *t* test). **e** Detection of HDAC enzymatic activity in HuCCT1 cells with vector or PFKL overexpression (n=3 biological replicates, Student’s *t* test). All the statistical data are presented as the means±SEMs.

**Supplementary Fig. 8**


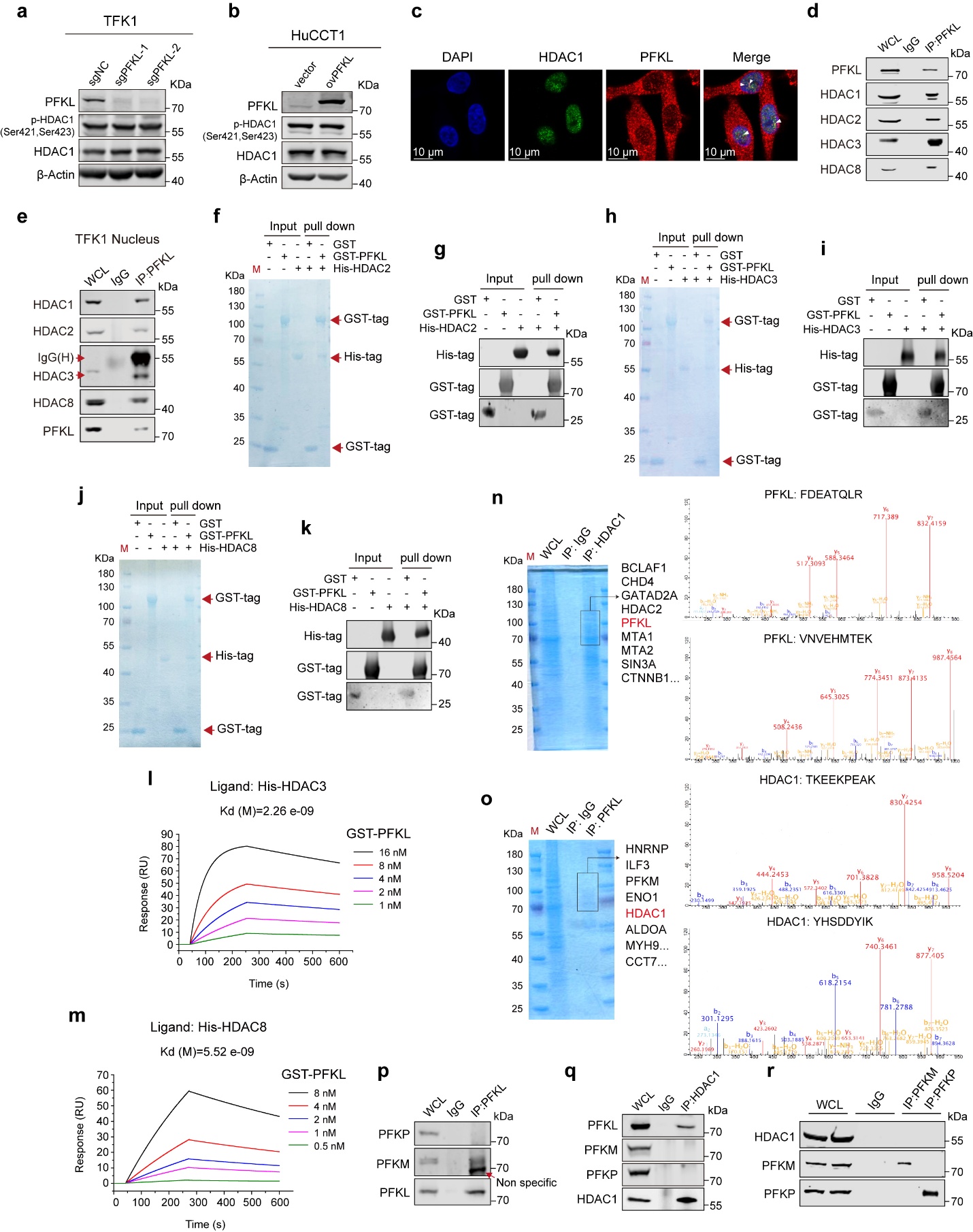


**Supplementary Fig. 8. PFKL directly binds to class Ⅰ HDACs. a** Immunoblot analyses of the expression of HDAC1, p-HDAC1, and PFKL in TFK1 cells expressing sgNC or sgPFKL. **b** Immunoblot analyses of the expression of HDAC1, p-HDAC1, and PFKL in HuCCT1 cells with vector or PFKL overexpression. **c** IF images of PFKL and HDAC1 staining in TFK1 cells. Scale bars, 10 μm. **d** Co-IP showing the interaction between endogenous PFKL and class Ⅰ HDACs in TFK1 cells. **e** Co-IP showing the interaction between endogenous PFKL and class Ⅰ HDACs in the TFK1 nucleus. **f, g** Coomassie brilliant blue staining and immunoblot analysis of a GST pull-down assay illustrating the interaction between GST-PFKL and His-HDAC2. Red arrows indicate corresponding bands. **h, i** Coomassie brilliant blue staining and immunoblot analysis of a GST pull-down assay illustrating the interaction between GST-PFKL and His-HDAC3. Red arrows indicate corresponding bands. **j, k** Coomassie brilliant blue staining and immunoblot analysis of a GST pull-down assay illustrating the interaction between GST-PFKL and His-HDAC8. Red arrows indicate corresponding bands. **l, m** The binding affinity detected by surface plasmon resonance (SPR) between PFKL and His-HDAC3 or His-HDAC8. **n** MS analysis was used to detect potential proteins that interact with HDAC1 in TFK1 cells. **o** MS analysis was used to detect potential proteins that interact with PFKL in TFK1 cells. **p** Co-IP showing the interaction between endogenous PFKL and PFKM or PFKP in TFK1 cells. **q, r** Co-IP showing the interaction between endogenous HDAC1 and PFK isoforms in TFK1 cells.

**Supplementary Fig. 9**


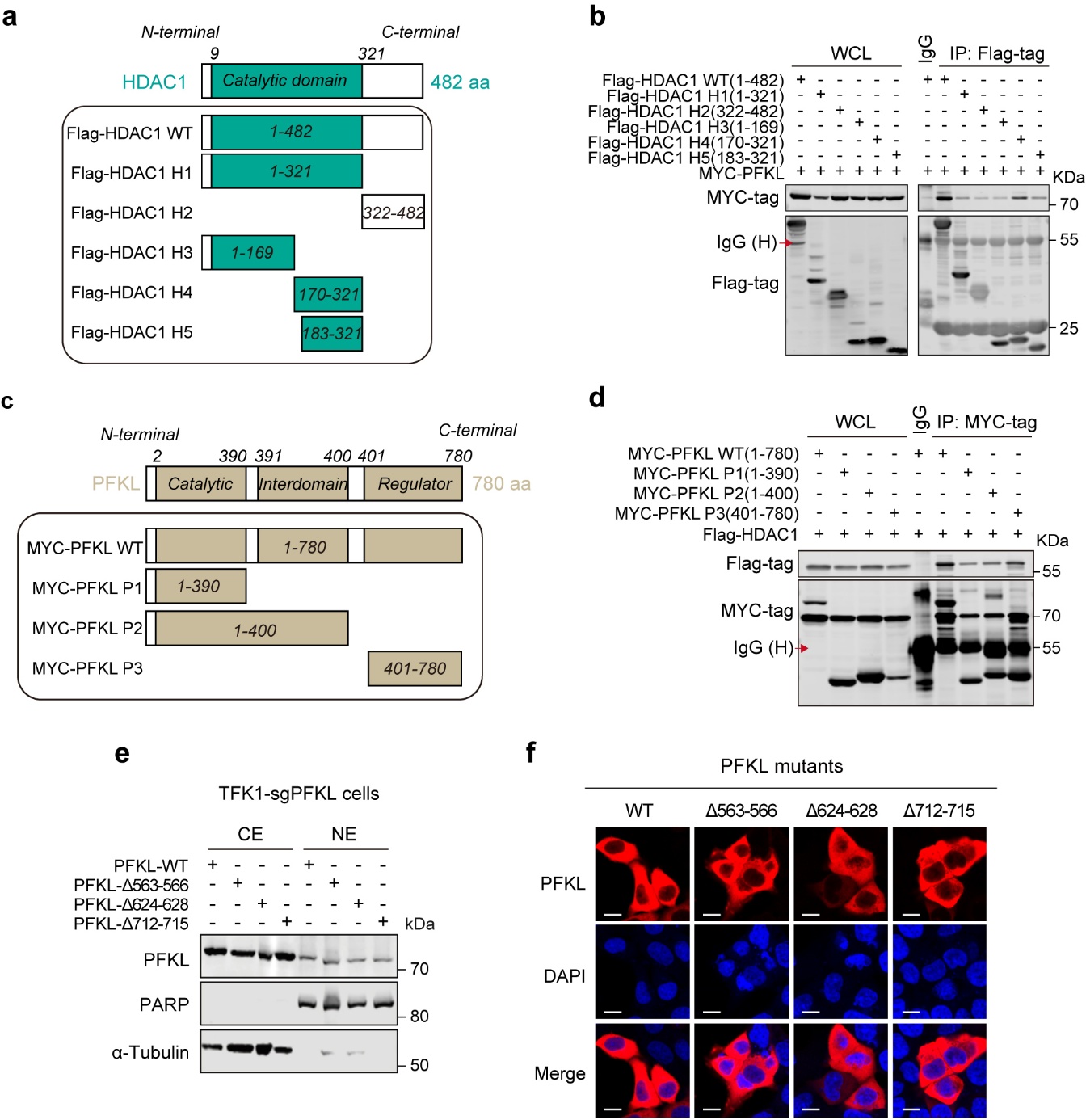


**Supplementary Fig. 9. The aa 170-183 region of HDAC1 mediates binding with the C-terminal regulatory domain of the PFKL protein. a** Schematic map of the construction of full-length and truncated HDAC1. **b** Co-IP showing the interactions between MYC-tagged PFKL and Flag-tagged full-length or truncated HDAC1 in HEK293T cells. **c** Schematic map of the construction of full-length and truncated PFKL. **d** Co-IP showing the interactions between Flag-tagged HDAC1 and MYC-tagged full-length or truncated PFKL in HEK293T cells. **e** Immunoblot analyses of the expression of PFKL in the nuclear extract (NE) and cytoplasmic extract (CE) of TFK1-sgPFKL cells transfected with PFKL-WT, PFKL-△563-566, PFKL-△624-628 and PFKL-△712-715 plasmids. **f** Representative IF images of PFKL and DAPI in TFK1-sgPFKL cells transfected with PFKL-WT, PFKL-△563-566, PFKL-△624-628 and PFKL-△712-715 plasmids. Scale bars, 10 μm.

**Supplementary Fig. 10**


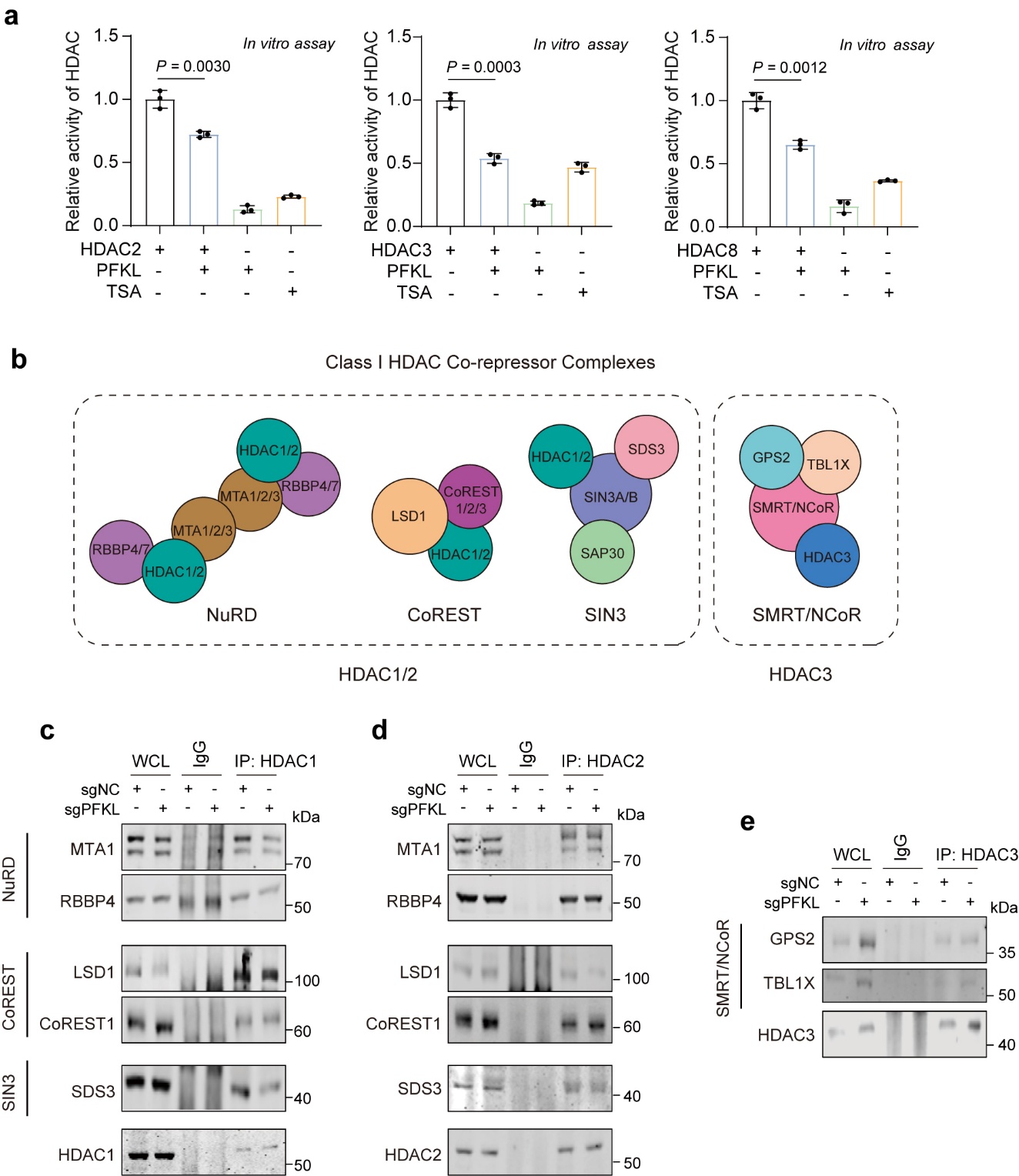


**Supplementary Fig. 10. PFKL inhibits the activity of class I HDAC proteins. a** In vitro enzymatic activity of purified Flag-tagged human HDAC2, HDAC3 and HDAC8 proteins from HEK-293T cells following incubation with purified PFKL. (n=3 biological replicates, Student’s *t* test). **b** Schematic representation of class I HDAC corepressor complexes. **c-e** Co-IP showing the interactions between class I HDACs and complex molecules in TFK1-sgNC and TFK1-sgPFKL cells. All the statistical data are presented as the means±SEMs.

**Supplementary Fig. 11**

**
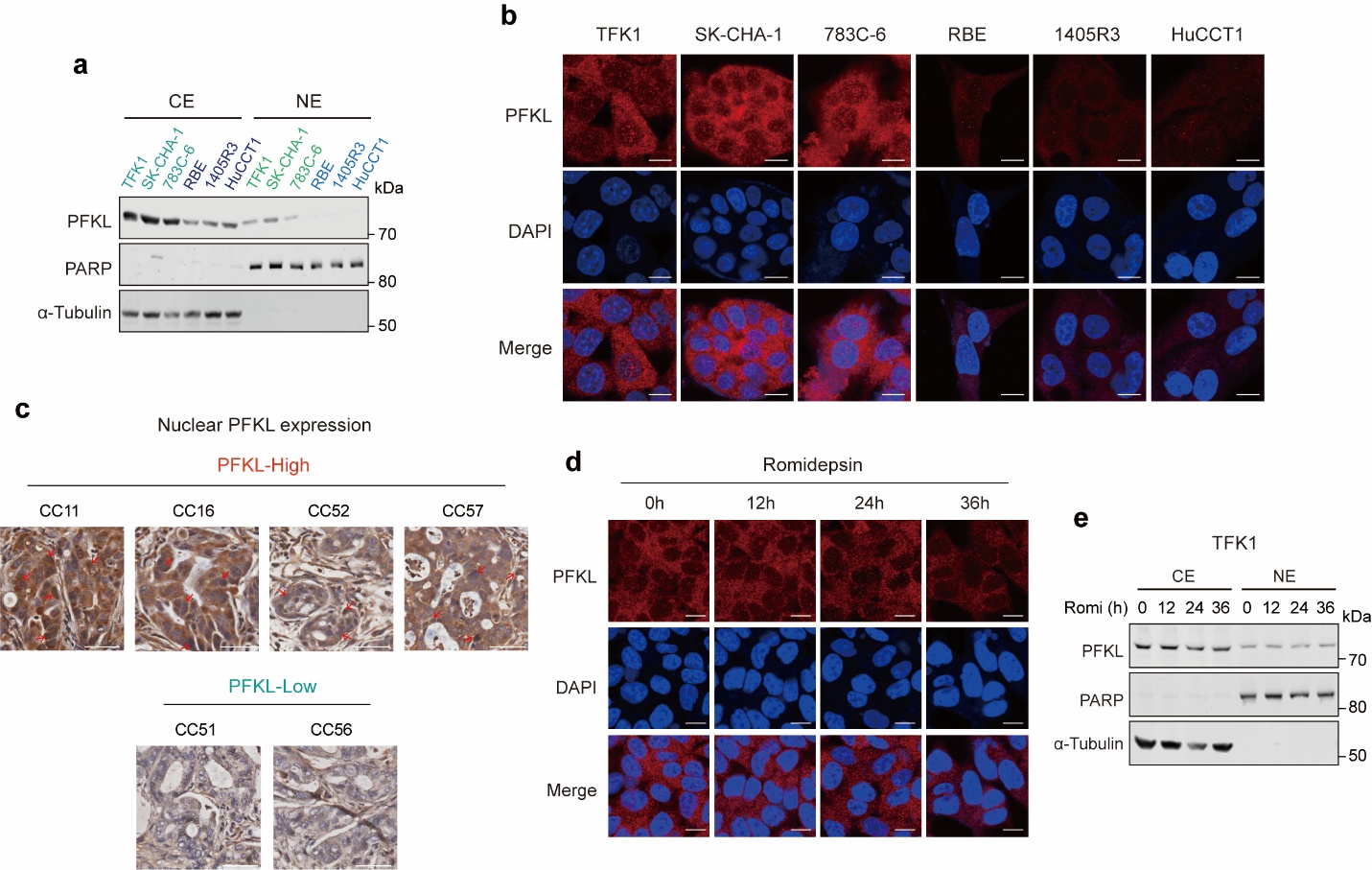
**

**Supplementary Fig. 11. Nuclear PFKL expression significantly regulates the efficacy of romidepsin. a** Immunoblot analyses of the expression of PFKL in the nuclear extract (NE) and cytoplasmic extract (CE) of different CCA cell lines. **b** Representative IF images of PFKL and DAPI in different CCA cells. Scale bars, 10 μm. **c** Representative images of nuclear PFKL staining in CCA PDX samples. The samples were categorized into “PFKL-High” or “PFKL-Low” groups on the basis of their nuclear PFKL staining intensity. Scale bars, 100 μm. **d** Representative IF images of PFKL and DAPI in TFK1 cells following treatment with specified concentrations of romidepsin for different durations. Scale bars, 10 μm. **e** Immunoblot analyses of the expression of PFKL in the CEs and NEs of TFK1 cells following treatment with romidepsin (10 nM) for different durations.

**Supplementary Fig. 12**

**
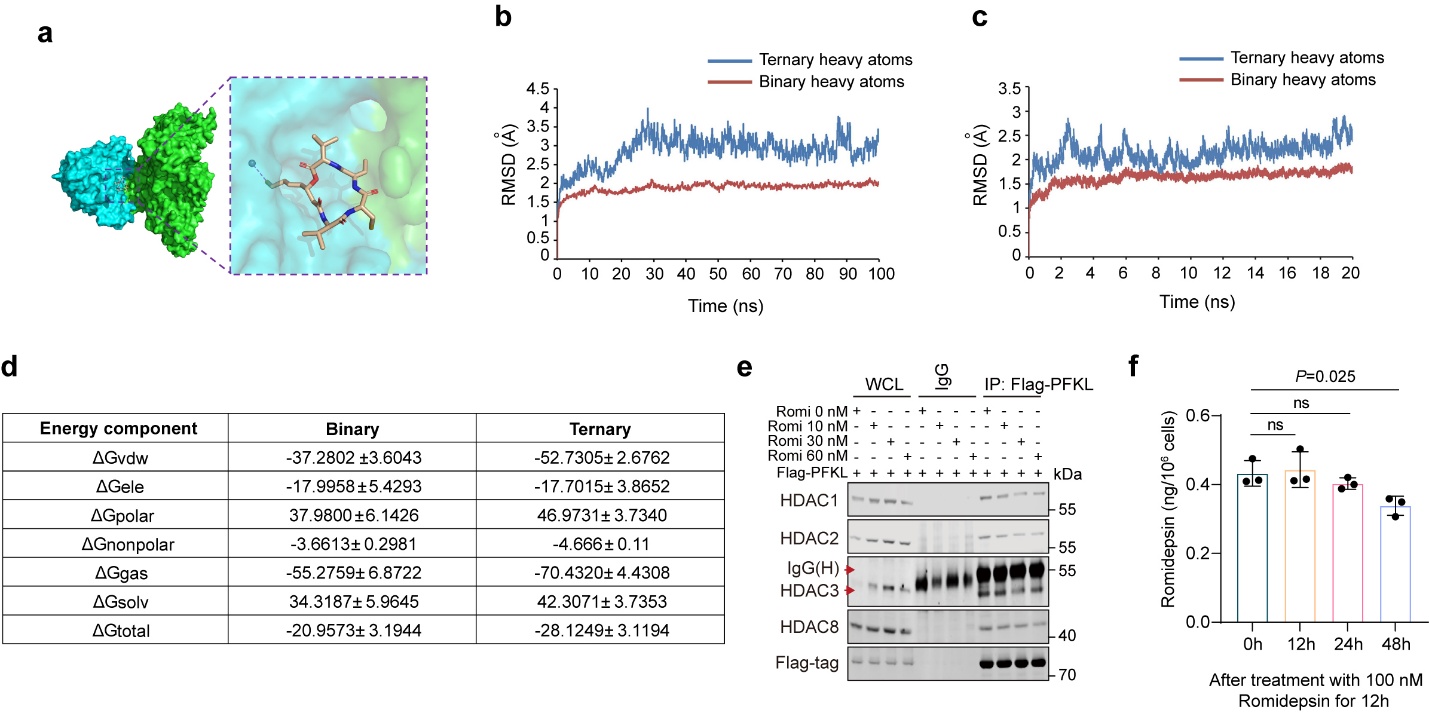
**

**Supplementary Fig. 12. PFKL enhances the affinity of romidepsin for chelation of zinc in HDACs. a** Molecular docking scheme of the ternary complex. HDAC1 is depicted as a cyan surface, and PFKL is represented by a green surface. Reduced-romidepsin is displayed as wheat-colored sticks, and zinc is presented as gray spheres. **b, c** Root mean square deviation (RMSD) curves of heavy atoms for binary and ternary complexes. Molecular dynamics simulations of 100 ns (B) and 20 ns (C) were conducted for both binary and ternary complexes. The binary complex is represented in red, and the ternary complex is shown in blue. **d** The binding free energies of binary and ternary complexes were calculated via the MM-PBSA method. **e** Coimmunoprecipitation showing the interaction between FLAG-tagged PFKL and class I HDACs in TFK1 cells treated with different concentrations of romidepsin. **f** Intracellular abundance of romidepsin detected in TFK1 cells after treatment with 100 nM romidepsin for 12 h (n=3 biological replicates, Student’s *t* test). All the statistical data are presented as the means±SEMs.

**Supplementary Fig. 13**


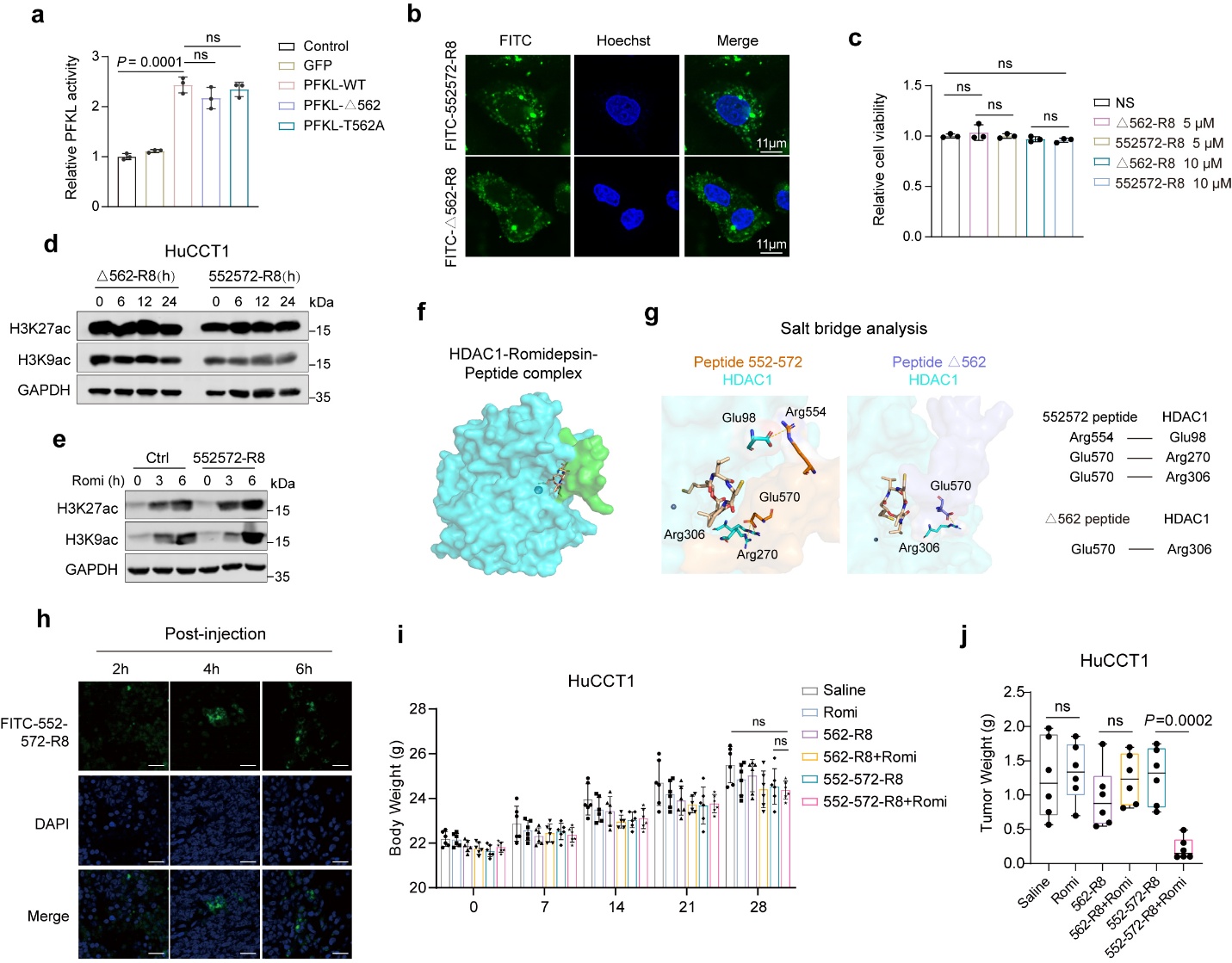


**Supplementary Fig. 13. The PFKL-552-572-R8 peptide promotes chelation effects between the ZBG of romidepsin and zinc within class** I **HDACs. a** Relative PFKL activity of HEK293T cells transfected with wild-type PFKL (PFKL-WT), PFKL-△562 or PFKL-T562A (n=3 biological replicates, one-way ANOVA). **b** Representative confocal images showing the infiltration of 552-572-R8 and △562-R8 in HuCCT1 cells. Scale bars, 11 μm. **c** The cytotoxic effects of the peptide on HuCCT1 cells were assessed via a CCK-8 assay (n=3 biological replicates, one-way ANOVA). **d** Immunoblot analyses of the expression of H3K9ac and H3K27ac in HuCCT1 cells following treatment with 552-572-R8 or △562-R8 for different durations. **e** Immunoblot analyses of the expression of H3K9ac and H3K27ac in HuCCT1 cells following treatment with specified concentrations of romidepsin combined with 552-572-R8. **f** Molecular docking scheme of the HDAC1-reduced-romidepsinromidepsin-peptide complex. HDAC1 is depicted as a cyan surface, and the peptide is represented by a green surface. Reduced-romidepsin is displayed as wheat-colored sticks, and zinc is presented as gray spheres. **g** Structural diagrams of protein-peptide interactions. HDAC1 is shown as a blue-green (cyan) cartoon (cartoon); 552-572-R8 and △562-R8 are shown in orange and dark blue-gray (slide) cartoons, respectively; reduced-romidepsin is shown as a wheat-colored (wheat) stick; the zinc ion is shown as a gray sphere; key residues are presented as sticks; yellow dashed lines indicate salt-bridging. **h** Representative confocal images of tumor cryosections demonstrating the penetration and distribution of the FITC-552-572-R8 peptide in HuCCT1 subcutaneous tumor tissues after intraperitoneal injection. Scale bars, 100 μm. **i** Body weight of each group of HuCCT1 xenografts at the end of treatment is plotted (n = 6 mice per group, two-way ANOVA). **j** The tumor weight of each group of HuCCT1 xenografts at the end of treatment is plotted (n = 6 mice per group, Student’s *t* test). All the statistical data are presented as the means±SEMs.

**Supplementary Fig. 14**


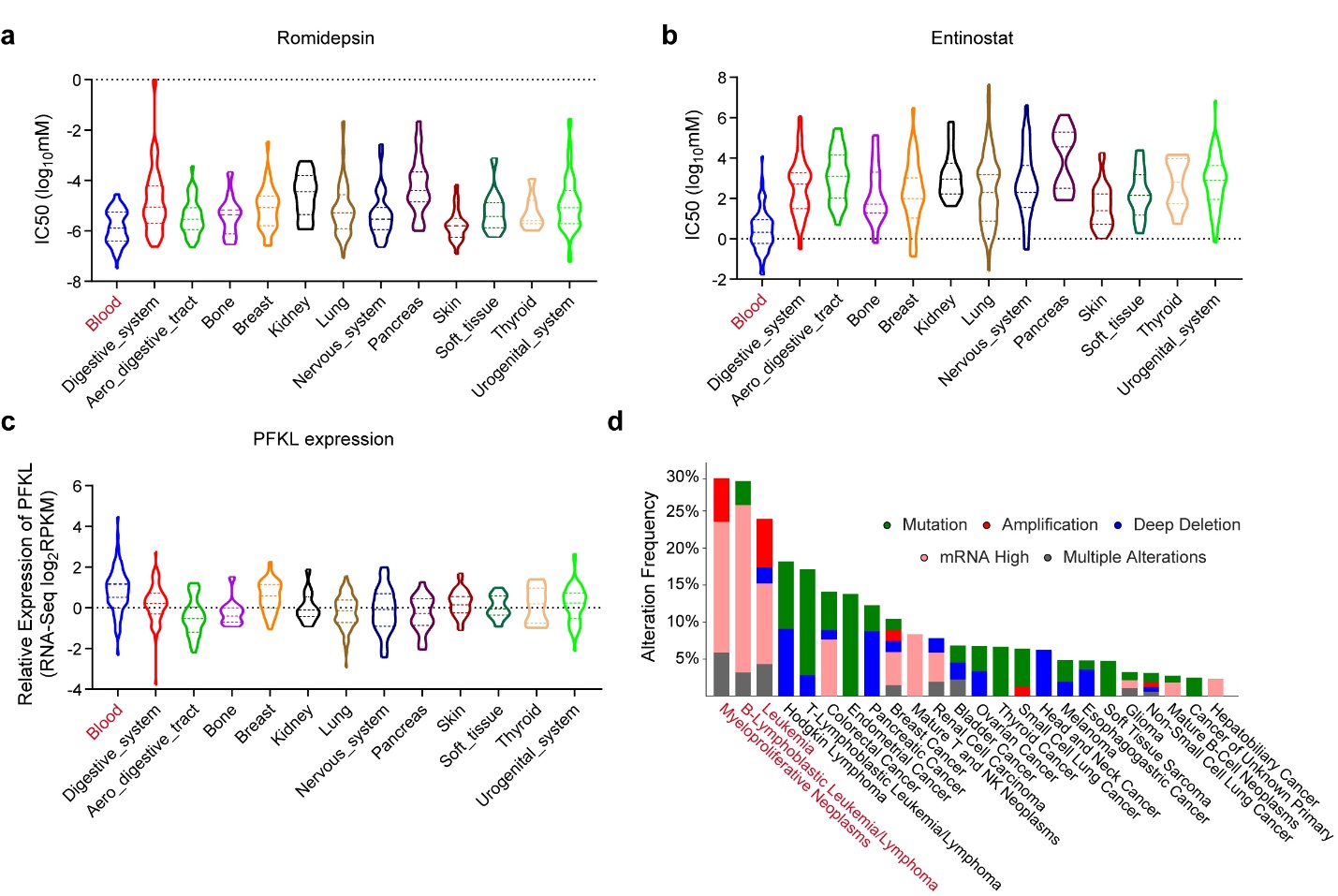


**Supplementary Fig. 14. PFKL enhances the effectiveness of HDACi across different cancer cells. a** The efficacy of romidepsin in tumor cells from different tissues in the GDSC database. **b** Efficacy of entinostat in tumor cells from different tissues in the GDSC database. **c, d** PFKL mRNA expression and genetic information of tumor cell lines in the Cancer Cell Line Encyclopedia (CCLE) database.

**Supplementary Fig. 15**


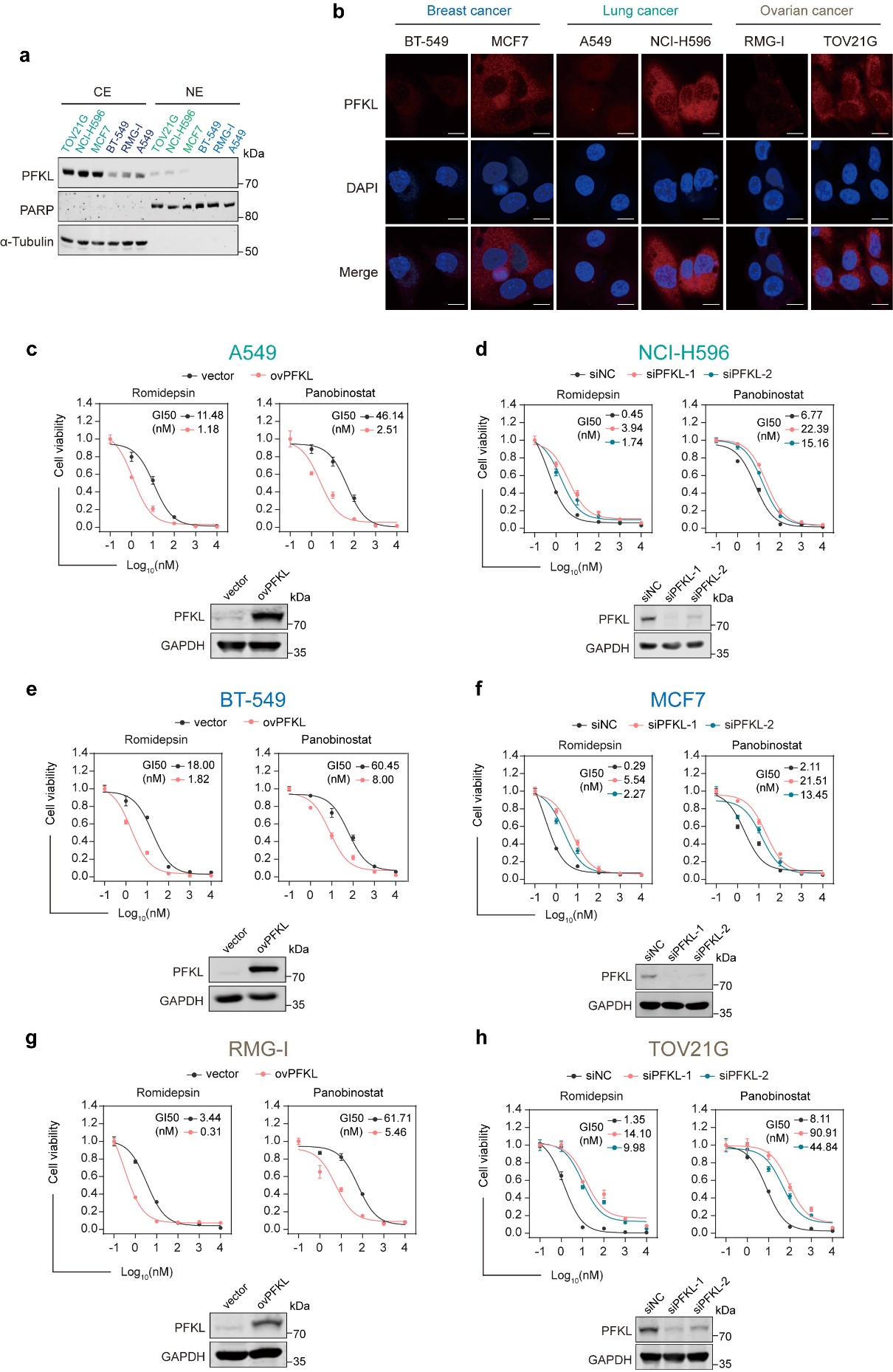


**Supplementary Fig. 15. The expression of nuclear PFKL regulates the efficacy of romidepsin in solid tumor cells. a** Immunoblot analyses of the expression of PFKL in the nuclear extract (NE) and cytoplasmic extract (CE) of different solid tumor cells. **b** Representative IF images of PFKL and DAPI in different solid tumor cells. **c** Cell viability of A549 cells with vector or PFKL overexpression after 72 hours of treatment with the indicated concentrations of romidepsin or panobinostat. **d** Cell viability of NCI-H596 cells with NC or PFKL siRNA after 72 hours of treatment with the indicated concentrations of romidepsin or panobinostat. **e** Cell viability of BT-549 cells with vector or PFKL overexpression after 72 hours of treatment with the indicated concentrations of romidepsin or panobinostat. **f** Cell viability of MCF7 cells with NC or PFKL siRNA after 72 hours of treatment with the indicated concentrations of romidepsin or panobinostat. **g** Cell viability of RMG-I cells with vector or PFKL overexpression after 72 hours of treatment with the indicated concentrations of romidepsin or panobinostat. **h** Cell viability of TOV21G cells transfected with NC or PFKL siRNA after 72 hours of treatment with the indicated concentrations of romidepsin or panobinostat.

**Supplementary Fig. 16**


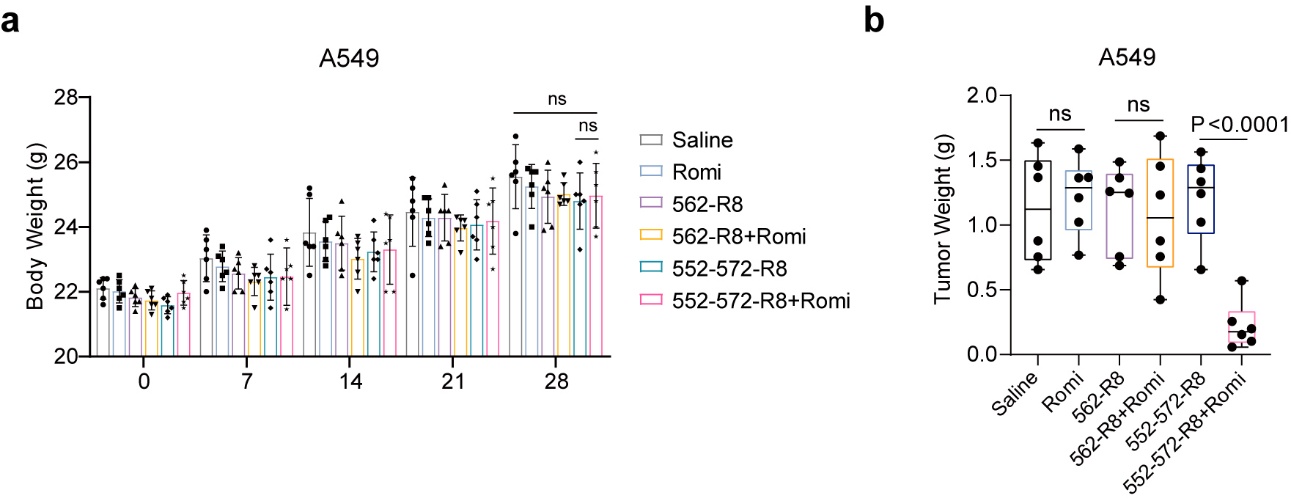


**Supplementary Fig. 16. The PFKL-552-572-R8 peptide promotes the efficacy of romidepsin in A549 tumors.** **a** Body weight of each group of A549 xenografts at the end of treatment is plotted (n = 6 mice per group, two-way ANOVA). **b** The tumor weight of each group of A549 xenografts at the end of treatment was plotted (n = 6 mice per group, Student’s *t* test). All the statistical data are presented as the means±SEMs.

**Supplementary Table 1**

Drug library

**Supplementary Table 2**

783C-6 DSRT analysis

**Supplementary Table 3**

1405R3 DSRT analysis

**Supplementary Table 4**

sgPFKL_Romi vs sgNC_Romi (H3K9ac) different peaks annotation

**Supplementary Table 5**

sgPFKL_Romi vs sgNC_Romi (H3K27ac) different peaks annotation

**Supplementary Table 6**

Mass spectrometry identification results of HDAC1 and PFKL

**Supplementary Table 7**

Salt bridge interaction between HDAC1 and peptides

**Supplementary Table 8**

CRISPR Screen data (TFK1-Romidepsin)

**Supplementary Table 9**

CRISPR Screen data (TFK1-Panobinostat)

**Supplementary Table 10**

CRISPR Screen data (783C-6-Romidepsin)

**Supplementary Table 11**

CRISPR Screen data (783C-6-Panobinostat)

**Supplementary Table 12**

qPCR primers

**Supplementary Table 13**

siRNA, shPFKL, sgPFKL sequences
